# Design, Synthesis, and Biological Evaluation of N14-Amino Acid-Substituted Tetrandrine Derivatives as Potential Antitumor Agents against Human Colorectal Cancer

**DOI:** 10.3390/molecules27134040

**Published:** 2022-06-23

**Authors:** Yu-Chan Wang, Rong-Hong Zhang, Sheng-Cao Hu, Hong Zhang, Dan Yang, Wen-Li Zhang, Yong-Long Zhao, Dong-Bing Cui, Yong-Jun Li, Wei-Dong Pan, Shang-Gao Liao, Meng Zhou

**Affiliations:** 1 State Key Laboratory of Functions and Applications of Medicinal Plants, Engineering Research Center for the Development and Application of Ethnic Medicine and TCM (Ministry of Education), Guizhou Medical University, Guiyang 550004, China; wyc1026508@163.com (Y.-C.W.); zhangrhla@163.com (R.-H.Z.); hushengcao0221@163.com (S.-C.H.); liyongjun026@126.com (Y.-J.L.); 2 Center for Tissue Engineering and Stem Cell Research, Key Laboratory of Regenerative Medicine of Guizhou Province, School of Basic Medical Sciences, Guizhou Medical University, Guiyang 550004, China; treebing@126.com; 3 School of Pharmacy, Guizhou Medical University, Guian New Districtc, Guiyang 550025, China; zh1134036410@163.com (H.Z.); yd1313132022@163.com (D.Y.); zhangwenli0717@163.com (W.-L.Z.); zhaoyonglong@gmc.edu.cn (Y.-L.Z.); lshangg@163.com (S.-G.L.)

**Keywords:** tetrandrine derivatives, amino acid, human colorectal cancer cell, autophagy, anti-angiogenesis

## Abstract

As a typical dibenzylisoquinoline alkaloid, tetrandrine (TET) is clinically used for the treatment of silicosis, inflammatory pulmonary, and cardiovascular diseases in China. Recent investigations have demonstrated the outstanding anticancer activity of this structure, but its poor aqueous solubility severely restricts its further development. Herein, a series of its 14-*N*-amino acid-substituted derivatives with improved anticancer effects and aqueous solubility were designed and synthesized. Among them, compound **16** displayed the best antiproliferative activity against human colorectal cancer (HCT-15) cells, with an IC_50_ value of 0.57 μM. Compared with TET, **16** was markedly improved in terms of aqueous solubility (by 5-fold). Compound **16** significantly suppressed the colony formation, migration, and invasion of HCT-15 cells in a concentration-dependent manner, with it being more potent in this respect than TET. Additionally, compound **16** markedly impaired the morphology and motility of HCT-15 cells and induced the death of colorectal cancer cells in double-staining and flow cytometry assays. Western blot results revealed that **16** could induce the autophagy of HCT-15 cells by significantly decreasing the content of p62/SQSTM1 and enhancing the Beclin-1 level and the ratio of LC3-II to LC3-I. Further study showed that **16** effectively inhibited the proliferation, migration, and tube formation of umbilical vein endothelial cells, manifesting in a potent anti-angiogenesis effect. Overall, these results revealed the potential of **16** as a promising candidate for further preclinical studies.

## 1. Introduction

The burden of cancer incidence and mortality is growing rapidly worldwide [1], and more than 19.3 million new cases and 10 million cancer deaths were estimated according to GLOBOCAN 2020 [2]. Colorectal cancer, also known as large bowel cancer, is one of the leading cause of cancer-related morbidity and mortality [3]. It is very heterogeneous, with a high variability in terms of patient prognosis and treatment response [4]. Approximately half of patients with colorectal cancer eventually succumb to the disease after the surgical resection of the primary tumor, predominantly due to metastases [5]. Therefore, metastasis is the main cause of colorectal cancer death, with it sometimes having already occurred even before the detection of the primary tumor [6]. So far, the molecular mechanism of metastasis remains poorly understood, but it is clear that the progression of metastasis involves the intravasation of cancer cells into blood vessels through basement membranes and migration to distant organ sites [7]. Migration, invasion, and angiogenesis are believed to play key roles in the metastasis of colorectal cancer [8,9,10,11]. Therefore, it is extremely urgent to develop novel agents to suppress the metastasis of colorectal cancer, especially for patients with unresectable tumors.

**Figure 1:**
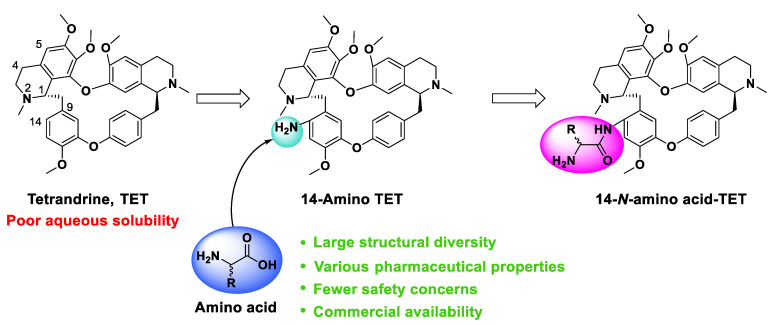
Design strategy of amino acid derivatives of TET.

The diversity and complexity of natural products afford remarkable efficacy in the discovery of anticancer agents, and thousands of natural compounds have been reported with anticancer properties [12,13,14,15,16]. Tetrandrine (TET, Figure 1) is a bisbenzylisoquinoline alkaloid that was originally isolated from the traditional Chinese medicinal plant *Stephania tetrandra* S. Moreover, multiple biological activities have been proposed for this structure, such as anti-inflammation, antiallergic, immunomodulation, heart-protective, and antihypertension effects [17,18]. TET is used in clinical contexts for the treatment of silicosis, autoimmune disorders, inflammatory pulmonary, and cardiovascular diseases [19]. Moreover, TET has been confirmed to have antiproliferative activity against various cancer cell lines, including hepatoma [17,18], breast cancer [20], lung carcinoma [21], leukemia [22], and prostate cancer [23]. Specifically, TET effectively inhibited the growth of human colorectal cancer both in vitro and in vivo [24,25], indicating its potential in the treatment of this disease. Molecular docking studies revealed that TET directly bound to the active domain of the orphan nuclear receptor 4A1 of pancreatic cancer cells [26], potentially interacted with the Akt of endometrial cancer cells [27] and the Protein kinase C-α (PKC-α) [28], and inhibited the ATP-binding cassette transporters of colon cancer cells [29]. However, the poor aqueous solubility of TET severely restricts its application [30], and structural optimizations on this agent are still required to improve its druggability.

To date, few structural modifications have been performed on TET, with these mainly focusing on the C_5_ and C_14_ positions, and experimental studies have shown that optimizations on the C_14_ position generally provides derivatives with better activities than TET against several cancer cells [31,32,33,34,35]. Nevertheless, fewer investigations on the enhancement of the solubility of TET have been reported, and most of the derivatives achieved the improvement through the nano-encapsulation strategy [36,37,38]. Large polymer amounts are required in these pharmaceutical applications, and these strategies cannot improve the druggability of TET itself. Natural and synthetic amino acids, which are commercially available to medicinal chemists [39], provide wide structural diversity and physicochemical properties in drug development [40]. Amino acids are excellent moieties with superior aqueous solubility and permeability, and a number of studies have successfully increased the solubility of chemical entities with the aid of amino acid fragments [41,42,43,44,45]. Several drugs (such as valacyclovir and valganciclovir) were also reported to have improved pharmaceutical properties by using these moieties [46,47,48]. In addition, as the building blocks of proteins, amino acids are generally regarded as safe molecular fragments. However, the introduction of most amino acid moieties increases the challenge of synthesis and purification because of the addition of an additional chiral center in the molecule [49]. Furthermore, some amino acids, such as L-glutamic acid and L-cysteine, have the potential to be converted into other amino acids (L-pyroglutamic acid and dimer cysteine, respectively) in solution, and moderate reaction conditions should be cultivated to take this into account [50]. Thus, in this study, a series of amino acids were attached to the C14 amino of 14-amino TET with a mild reaction condition (Figure 1), which was derived from TET with a moderate anticancer effect [32], with the aim of improving its solubility and biological activity. Encouragingly, consistent with the above hypothesis, a number of TET derivatives were found with outstanding anticancer activities and aqueous solubilities.

## 2. Results and Discussion

### 2.1. Chemistry

**Scheme 1:**
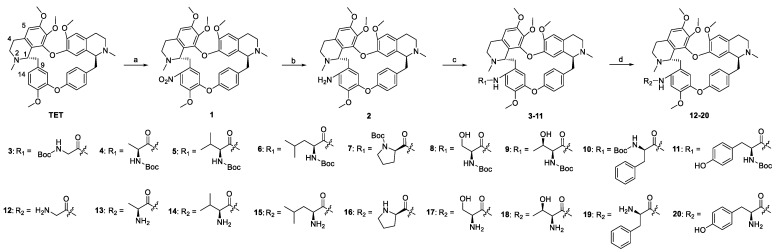
Synthesis of TET derivatives. (**a**) Mixed acid (HNO_3_:(CH_3_CO)_2_O = 3:5, *v*/*v*), CH_2_Cl_2_, −10 °C to 0 °C, 4 h, 92%. (**b**) SnCl_2_•2H_2_O, EtOAc, 80 °C, 3 h, 45%. (**c**) Boc-amino acid, DCC, CH_2_Cl_2_, rt, 3–4 h, 85–93%. (**d**). TFA, CH_2_Cl_2_, rt, 2 h, 85–91%.

The synthesis of TET derivatives (**3**–**20**) is depicted in Scheme 1. Since optimizations on the C_14_ position of TET have commonly provided derivatives with better anticancer activities than the parent compound [32,33], herein, a series of L-amino acids were attached to this position through an amide bond. The C_14_ position of TET was selectively nitrified at a mild condition to afford 14-nitro-TET (**1**) with a high yield, the nitro of which was then reduced to an amino group (**2**) to facilitate the introduction of amino acids. An amide condensation reaction then proceeded between compound **2** and nine Boc-protected amino acids to afford **3**–**11** in the presence of condensing agent *N*,*N*’-dicyclohexylcarbodiimide (DCC). Then, the N-protected groups of **3**–**11** were removed smoothly with trifluoroacetic acid (TFA) to provide derivatives **12**–**20**. All the synthesized compounds were characterized by IR, ^1^H NMR, ^13^C NMR, and high-resolution mass spectrum (HRMS).

### 2.2. Aqueous Solubility Analysis

**Figure 2:**
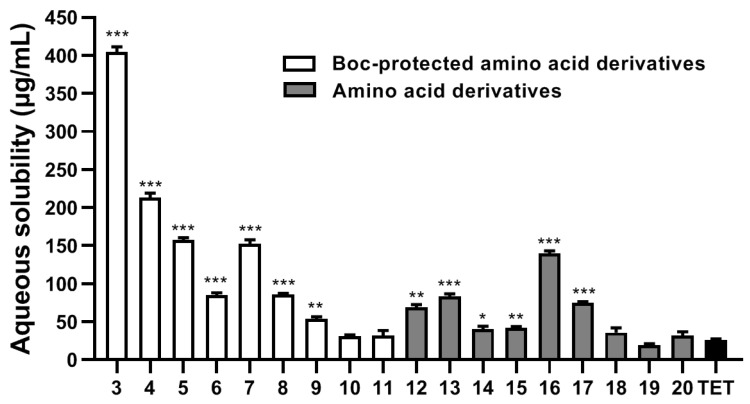
Aqueous solubility analysis of TET derivatives in neutral water. *: *p* < 0.05, **: *p* < 0.01, ***: *p* < 0.001 vs. TET.

To better understand the improvement in terms of solubility as a result of introducing L-amino acids, three polar amino acids (Ser, Thr, and Tyr) and six non-polar amino acids (Gly, Ala, Val, Leu, Pro, and Phe) were selected in this study, and the water-solubility of TET derivatives was measured by HPLC according a reported procedure [51]. As presented in Figure 2, the solubility of TET was very poor in water (less than 30 μg·mL^−1^), which was consistent with the reported study [52]. By contrast, except for **10**, **11**, and **18**–**20**, most of the amino acid derivatives exhibited higher solubilities than TET (*p* < 0.05). Most notably, the solubility of Boc-protected glycine derivative **3** significantly increased approximately 16-fold compared to TET. Among the amino acid derivatives, compound **16** displayed a marked improved aqueous solubility (>5-fold), indicating that the incorporation of amino acids can effectively improve the water solubility of TET.

### 2.3. Antiproliferative Activity

#### 2.3.1. Cytotoxicity Analysis

The antiproliferative effect of the synthesized TET derivatives was evaluated by MTT assay against five common cancer cell lines, including human lung cancer cells (A549), colon cancer cells (HCT-15), liver cancer cells (HepG2), pancreatic cancer cells (BxPC-3), and breast cancer cells (MCF-7). Meanwhile, the toxicity and anti-angiogenesis activity of the title compounds were evaluated with respect to human normal hepatocyte cells (L-02) and umbilical vein endothelial cells (HUVEC), respectively. TET was evaluated as a comparison. As shown in Table 1, except for **8** and **9**, most of the prepared compounds significantly inhibited the proliferation of cancer cells, with IC_50_ values comparable to or lower than that of TET. These TET derivatives exhibited better inhibitory activities on HCT-15, HepG2, and BxPC-3 than on A549 and MCF-7, and these derivatives displayed the best anticancer effect on HCT-15 cells, with IC_50_ values ranging from 0.57 to 5.28 μM. Specifically, compared with TET (IC_50_ = 6.12 μM), the introduction of Boc-protected glycine to the C_14_ amino of TET **3** improved the antiproliferative activity by about 2-fold against HCT-15 cells. Increasing the substituent size on the methylene of glycine, from methyl **4** to isopropyl **5** and isobutyl **6**, gradually impaired the inhibitory activity, which was most likely attributed to the reduction in terms of aqueous solubility. However, the attachment of Boc-protected proline to the C_14_ amino of TET **7** enhanced the anticancer effect (with an IC_50_ of 1.15 μM). By contrast, the introduction of Boc-protected serine **8** and threonine **9** markedly weakened the activity. Interestingly, for Boc-protected phenylalanine derivative of TET **10**, the antiproliferative effect was increased by more than 6-fold compared to TET, with IC_50_ decreased to 0.91 μM, whereas the Boc-protected tyrosine derivative **11** only exhibited a retained activity. 

The protecting groups of **3**–**11** were removed to provide derivatives **12**–**20,** respectively, with generally improved anticancer effects against HCT-15 cells (Table 1). The inhibitory activity trend of **12**–**20** were almost in line with the parent compounds **3**–**11**. Among them, the L-proline derivative **16** exhibited the best antiproliferative activity, and with IC_50_ values that increased to higher than 10-fold (0.57 μM) the values for TET. Moreover, compound **16** also effectively inhibited the proliferation of the other four cancer cells, with IC_50_s lower than 1.5 μM. Regrettably, no enhancement in terms of antiproliferative activity was observed for the deprotected product of **10** (**19**). 

Considering its improved aqueous solubility and anticancer activity, compound **16** was then selected for the further biological evaluation. Notably, during the cytotoxicity assay, the morphology of the HCT-15 cells was altered after the treatment of **16**, and cell shrinkage and nuclear karyorrhexis were observed, indicating the disorganization of the internal architecture (Figure S1 in Supplementary Materials). To determine the safety of the synthesized compounds, the toxicity of **16** was then evaluated on human hepatic cells L-02 (Table 1). Compared with their inhibitory activity against the cancer cells, lower cytotoxicities were observed for TET derivatives **3**–**20** on the normal cell, and the IC_50_ values of **16** were >20 μM, suggesting that the compound had a lower toxicity.

#### 2.3.2. Colony Formation Assay

**Figure 3:**
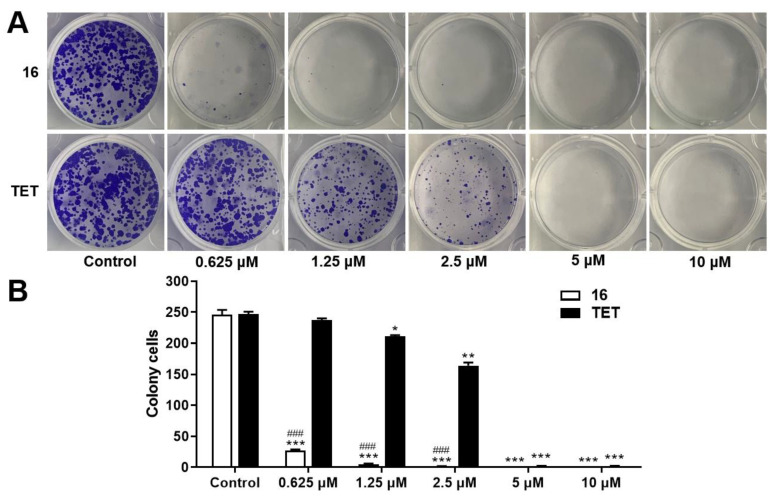
Inhibitory effect of **16** on the colony formation of HCT-15 cells. (**A**) HCT-15 cells were incubated with varying concentrations of **16** (0.625 1.25, 2.5, 5, and 10 μM) for 10 d, and the colonies were stained with crystal violet. (**B**) Quantifications of colony numbers. *: *p* < 0.05, **: *p* < 0.01, ***: *p* < 0.001, vs. control group; ^###^: *p* < 0.001, vs. TET at the same concentration.

The HCT-15 cell is a widely used cell line for studying the tumor biology and experimental therapy of human colorectal cancer in vitro [53], and this cell was used to investigate the anticancer effect of **16** in the following experiments. To evaluate the ability of **16** to undergo unlimited division and form colonies, the colony formation assay was performed on HCT-15 cells. As shown in Figure 3A, compound **16** effectively inhibited the colony formation of HCT-15 cells in a concentration-dependent manner, which was superior to TET at 1.25 and 2.5 μM (*p* < 0.05, Figure 3B). Specially, very few colonies were found in the plate treated with **16** even at 0.625 μM. These results indicated that compound **16** markedly inhibited the colony formation of HCT-15 cells.

#### 2.3.3. Migration Assay

**Figure 4:**
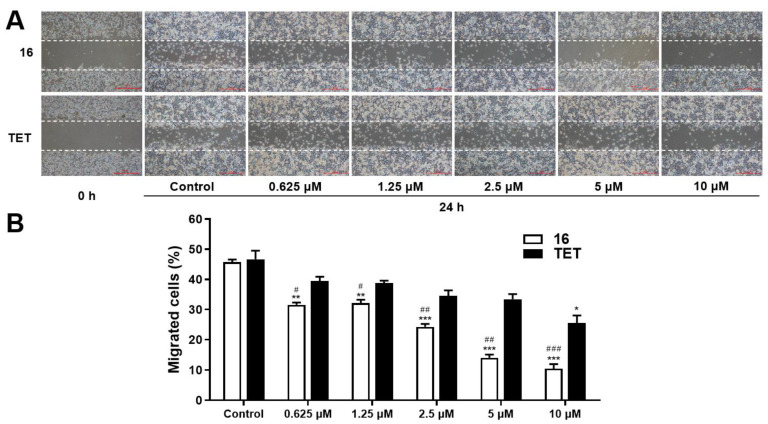
Effect of **16** on the migration of HCT-15 cells determined by wound healing assay. (**A**) Images of HCT-15 cell migration inhibited by **16** (0.625 1.25, 2.5, 5, and 10 μM). (**B**) Filled areas calculated by ImageJ software. *: *p* < 0.05, **: *p* < 0.01, ***: *p* < 0.001 vs. control group; ^#^: *p* < 0.05, ^##^: *p* < 0.01, ^###^: *p* < 0.001 vs. TET at the same concentration.

The capacity of **16** to inhibit migration, invasion, and angiogenesis was then assessed to investigate its antimetastatic potential. The wound healing assay was carried out to evaluate the effect of compound **16** on the migration of HCT-15 cells. The results revealed that the scratched areas in the control groups were approximately 46% covered after 24 h of culture (Figure 4A,B). However, the recovery of the scratched areas was suppressed with the treatment of **16** μM in a concentration-dependent manner (*p* < 0.01). Compared with TET, derivative **16** exhibited much lower migration rates at all the concentrations (*p* < 0.05). Specifically, the scratched areas decreased to 14.1% and 10.5% after the addition of 5 and 10 μM of **16**, respectively, indicating its strong suppressing effect on the migration of HCT-15 cells.

#### 2.3.4. Invasion Assay

**Figure 5:**
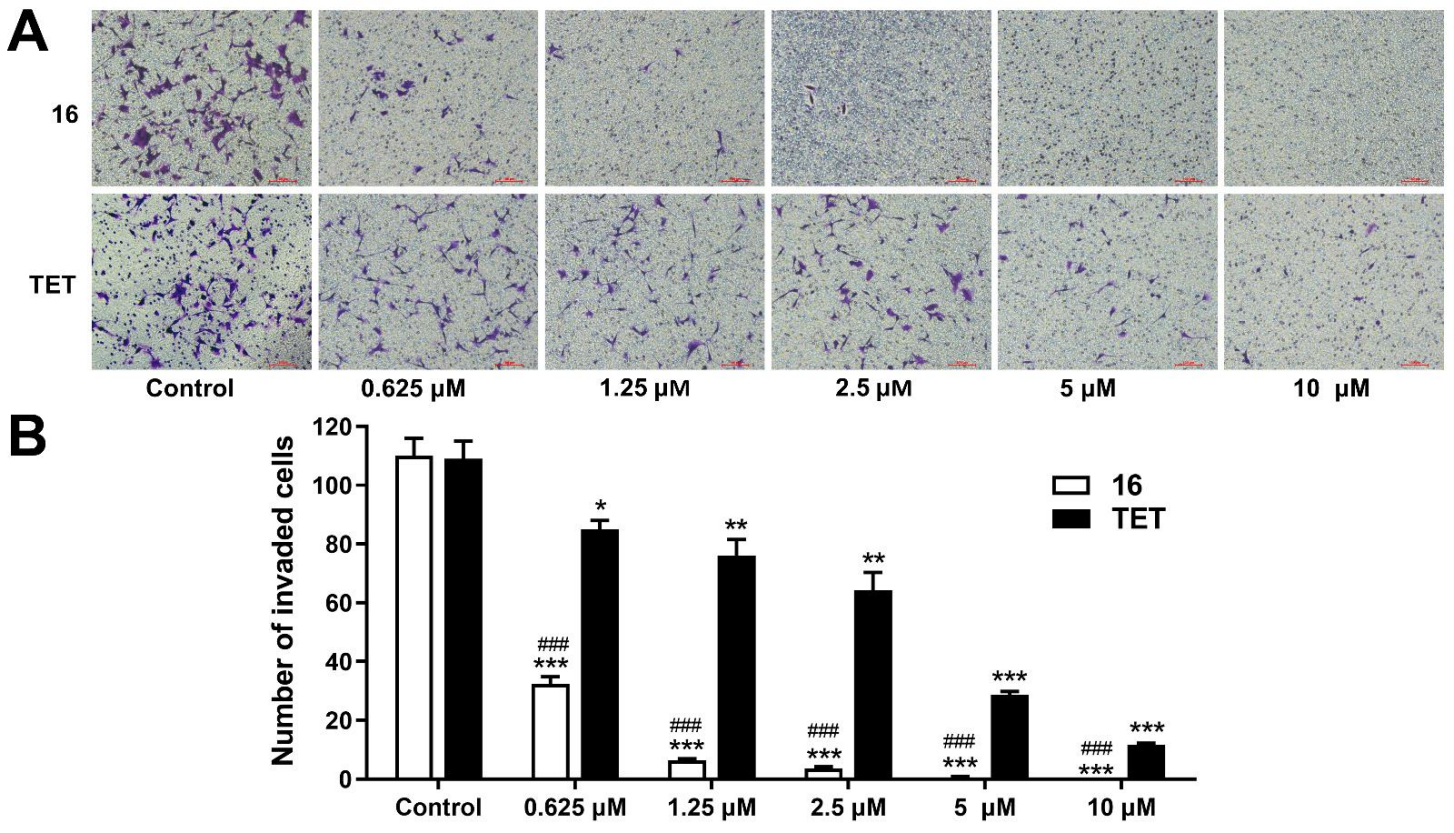
Invasion suppressing effect of **16** against HCT-15 cells by transwell assay. (**A**) Suppressing effect of **16** (0.625, 1.25, 2.5, 5, and 10 μM) on the invasion of HCT-15 cells. (**B**) Quantitative analysis of invaded cells. *: *p* < 0.05, **: *p* < 0.01, ***: *p* < 0.001, vs. control group; ^###^: *p* < 0.001 vs. TET at the same concentration.

During the metastasis of cancer, cell invasion through the extracellular matrix is a significant process, and the detached cancer cells can move to distant organs for metastasis [54]. To investigate the ability of **16** to inhibit invasion, a transwell assay was then carried out on HCT-15 cells. After the treatment of **16**, the invasions of colorectal cancer cells were significantly weakened in a concentration-dependent manner (Figure 5A), with the effect being more potent than that of TET at all the concentrations (*p* < 0.05, Figure 5B). Moreover, almost no cancer cells were observed for **16** at 5 and 10 μM.

#### 2.3.5. Morphological Analysis

**Figure 6:**
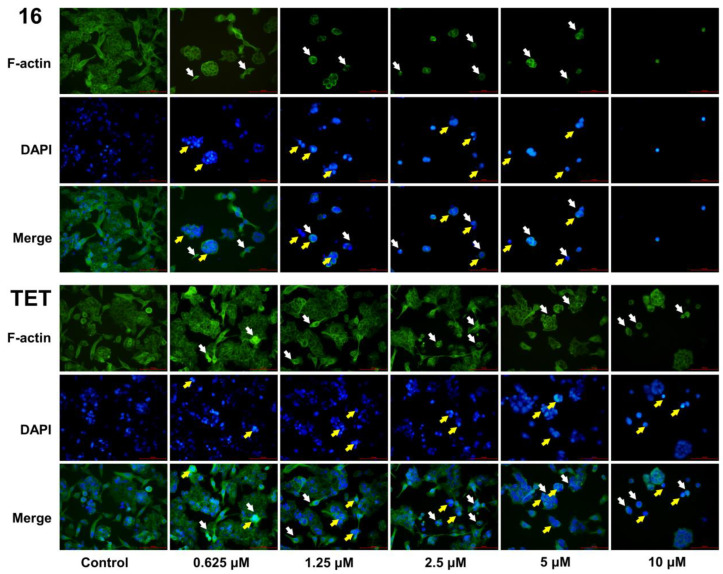
Morphological analysis of HCT-15 cells treated with **16** (0.625, 1.25, 2.5, 5, and 10 μM). F-actin proteins and nuclei were stained with FITC-phalloidin (green) and DAPI (blue), respectively. White arrows represent cytoskeleton disruption and yellow arrows nuclear damage.

Since the migration and invasion of HCT-15 cells were inhibited by **16**, the morphological changes in the cancer cells induced by this compound were further evaluated. The F-actin filaments and nuclei of HCT-15 cells were stained with Phalloidin-FITC and DAPI, respectively. Microfilaments are the major components which maintain the normal architecture of cells and play an important role in the motility, differentiation division, and membrane organization of cancer cells. As depicted in Figure 6, HCT-15 cells in the control group exhibited a regular array of F-actin filaments present along the cells. By contrast, a loss in cell volume and cell shrinkage were observed after treatment with **16** for 24 h, with these effects being more obvious than those in the TET groups. Additionally, the cells displayed a reduced amount of F-actin and a disorganization of actin filaments after the treatment of **16**, indicating that the cytoskeleton of HCT-15 cells was damaged and the motility significantly impaired. As a result, the migration and invasion of HCT-15 cells were severely suppressed. 

#### 2.3.6. Cell Death Analysis

**Figure 7:**
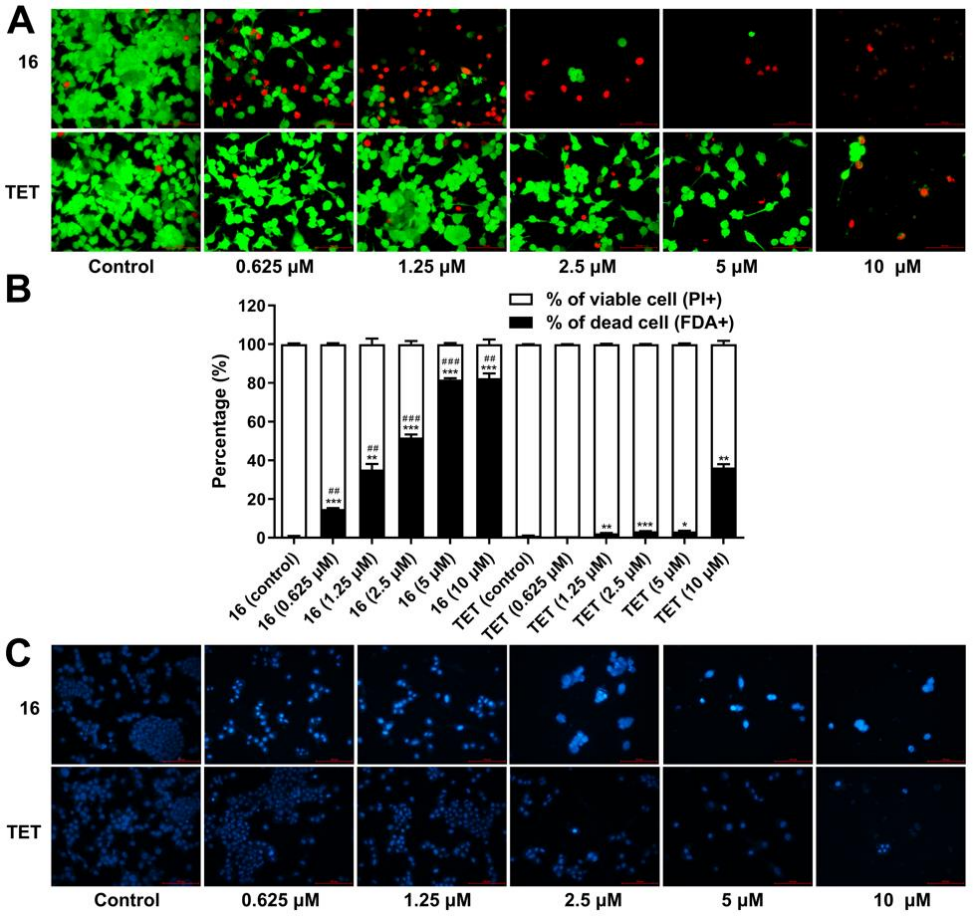
Effect of **16** on the induction of HCT-15 cell death. (**A**) Pictures of HCT-15 cells stained with FDA/PI. (**B**) Statistical analysis of death cells induced by **16** (0.625, 1.25, 2.5, 5, and 10 μM). (**C**) Nuclear changes of HCT-15 cells stained with Hoechst 33258. *: *p* < 0.05, **: *p* < 0.01, ***: *p* < 0.001, vs. control; ^##^: *p* < 0.01, ^###^: *p* < 0.001, vs. TET at the same concentration.

To better understand the cytotoxicity of compound **16** against HCT-15 cells, double staining with fluorescein diacetate (FDA)/propidium iodide (PI) was conducted to analyze the cell death induced by TET derivatives. Very few dead cells stained with PI (red) were detected in the control group (Figure 7A), whereas the amount of dead cells was markedly increased after the addition of **16**. Only a small number of vital cells that took up the fluorogen FDA (green) were observed for **16** at 5 and 10 μM. Statistical analysis revealed that the percentage of dead cells was enhanced with an increase in the concentration of **16** compared with the control group (*p* < 0.01, Figure 7B), and only 18.3% and 17.6% of viable cells were retained at 5 and 10 μM, respectively. Meanwhile, the cell death rate of **16** was much higher than that of TET at the same concentration (*p* < 0.05).

Hoechst 33258 staining was performed to investigate the effect of **16** on the nuclear morphology of colorectal cancer cells. As illustrated in Figure 7C, the nucleus of HCT-15 cells in the control groups were stained in blue and were uniform in shape. By contrast, the nuclear morphology of cancer cells was altered after treatment with **16**. The chromatin was obviously swollen in the nucleus of cancer cells incubated with **16**, with it being much brighter than in the control groups, particularly at concentrations higher than 1.25 μM. Moreover, nuclear fragmentation was also observed, indicating characteristics that differed from the typical apoptosis [55,56]. 

#### 2.3.7. Flow Cytometry Assay

**Figure 8:**
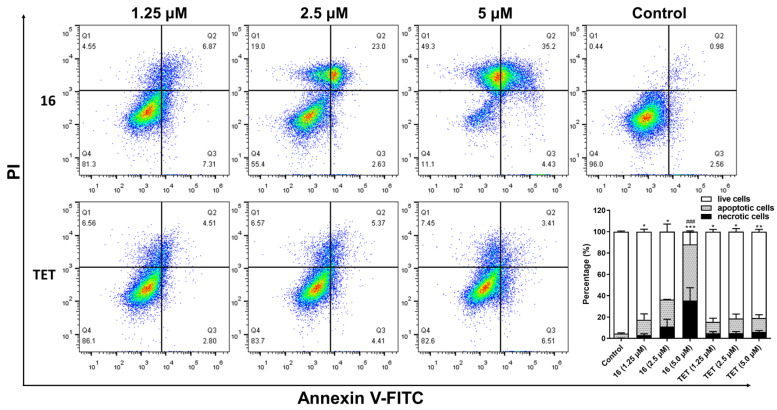
Flow cytometry analysis of apoptosis induction of **16** (1.25, 2.5, and 5 μM) in HCT-15 cells. Statistical analysis of the apoptosis is depicted in the bottom right panel. *: *p* < 0.05, **: *p* < 0.01, ***: *p* < 0.001 vs. control; ^###^: *p* < 0.001 vs. TET at the same concentration.

In order to investigate the characteristics of compound **16**, flow cytometry analysis was used to analyze cell apoptosis and the cell cycle. The results revealed that **16** effectively increased the apoptotic percentage of HCT-15 cells in a concentration-dependent manner (*p* < 0.05), with apoptotic rates of 25.4% and 52.7% in the case of **16** at concentrations of 2.5 and 5 μM, respectively (Figure 8). By contrast, only small percentages of apoptotic cells (lower than 14%) were detected in the TET treatment groups. Additionally, cell necrosis was also observed in the HCT-15 cells with the treatment of **16** at concentrations of 2.5 and 5 μM, with necrotic rates of 11.0% and 35.5%, respectively. Necrosis is commonly recognized as a side effect of anticancer agents [57]; however, as a genetically programmed form of necrotic cell death, necroptosis is also associated with the progression, metastasis, and immunosurveillance of cancer cells [58]. Thus, further study of this mechanism study is required to clarify the anticancer effect of **16**.

The effect of **16** on cell cycle progression was then investigated (Figure S2), but no significant influence on cell cycle redistribution was observed at concentrations of 1.25, 2.5, and 5 μM. These results suggested that, despite the induction of significant apoptosis in HCT-15 cells, the anticancer activity of **16** is independent of cell cycle control [59,60], and in this respect it diverges from commonly reported anticancer agents.

#### 2.3.8. Western Blot Analysis

**Figure 9:**
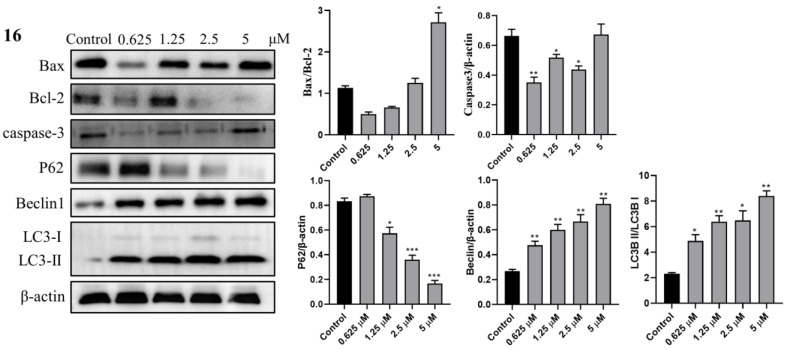
Effect of **16** on the expression of apoptotic (Bax, Bcl-2, and caspase-3) and autophagic related (p62, Beclin1, LC3-I, and LC3-II) proteins in HCT-15 cells. Quantitative histogram analysis of protein expression. *: *p* < 0.05, **: *p* < 0.01, ***: *p* < 0.001 vs. control group.

To further study the anticancer mechanism of **16** in HCT-15 cells, the expression of key apoptosis-related proteins (Bax, Bcl-2, and caspase-3) was investigated by Western blot analysis [61,62]. As shown in Figure 9, the ratio of Bax/Bcl-2 was enhanced (*p* < 0.05) only after treatment with a high concentration of **16** (5 μM). Additionally, compound **16** significantly decreased the caspase-3 level at 0.625, 1.25, and 2.5 μM (*p* < 0.05), and no concentration dependence was observed. These results indicated that the anticancer mechanism of **16** should be distinguished from typical apoptosis. 

Several investigations have demonstrated that TET induces autophagy in cancer cells [63,64]; thus, the autophagic related proteins p62/SQSTM1 (P62), Beclin-1, and microtubule-associated protein 1 light chain 3 (LC3) were further evaluated to identify the activity of **16** in terms of autophagy induction in HCT-15 cells. As depicted in Figure 9, P62 contents were significantly lessened (*p* < 0.05) after the treatment of **16** (1.25, 2.5, and 5 μM) in a concentration-dependent manner. By contrast, the levels of Beclin-1 were markedly enhanced with an increase in the concentration of **16** (*p* < 0.01). Also, the ratios of LC3-II to LC3-I were increased for **16** (0.625, 1.25, 2.5, and 5 μM, *p* < 0.05). The above results demonstrated that **16** effectively induced the death of HCT-15 cells through autophagy.

#### 2.3.9. Molecular Docking Study

**Figure 10:**
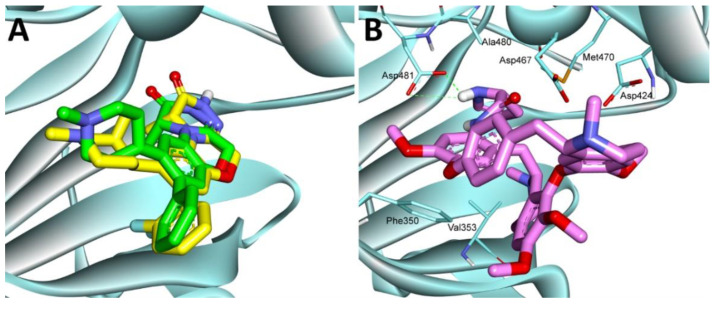
Binding mode analysis of **16**. (**A**) Overlay of co-crystallized **28** (green carbon) and re-docked **28** (yellow carbon) with PKC-α (PDB ID: 4RA4). (**B**) Binding mode of **16** (pink carbon) in complex with PKC-α. PKC-α is shown in cyan.

Molecular docking is a powerful tool which is used to predict the binding mode of a small molecule to a protein target, and several binding modes of TET have been reported so far. Among them, TET was proved to promote autophagy mediated-cell death in cancer cells by inhibiting PKC-α [28], which is consistent with the above observation in TET derivative **16**. Therefore, the binding pose of **16** with PKC-α (PDB ID: 4RA4) was investigated by Autodock 4.2. Beforehand, the PKC-α inhibitor 28 [65] was docked into the binding site in order to validate the docking method (Figure 10A). Results showed that the re-docked 28 (yellow carbon) was almost superimposed with that of the co-crystallized ligand (green carbon), indicating the reliability of the docking method. Then, **16** was docked into the active cavity. As depicted in Figure 10B, **16** was well anchored to the binding site of PKC-α and interacted with amino acid residues including Phe350, Val353, Met470, Asp467, Ala480, and Asp 481. Further structural analyses revealed that a pi–pi stacking interaction was observed between Phe350 and the phenyl group of **16**, and strong hydrophobic forces were also involved between **16** and Val353 and Ala480. Moreover, two strong hydrogen bonds were formed between the C_14_ proline and Asp 481, which was speculated as the main cause of the strong anticancer effect of **16**.

#### 2.3.10. Anti-Angiogenesis Activity Analysis

**Figure 11:**
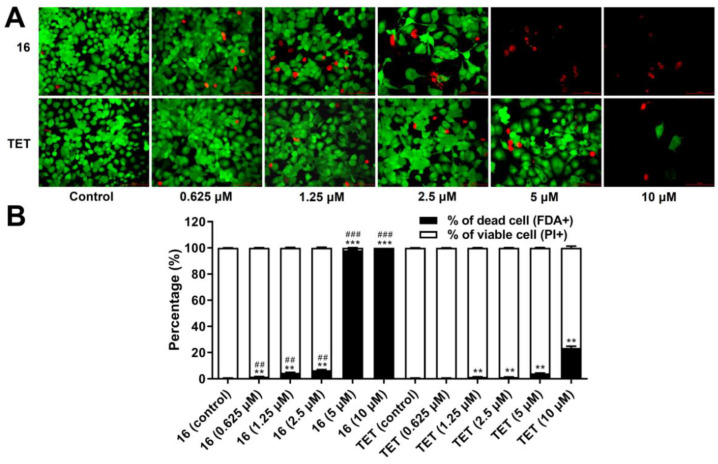
Effect of **16** on the cell death of HUVECs. (**A**) Figures of HUVECs stained with FDA/propidium PI. (**B**) Statistical analysis of dead cells induced by **16**. **: *p* < 0.01, ***: *p* < 0.001 vs. control; ^##^: *p* < 0.01, ^###^: *p* < 0.001 vs. TET at the same concentration.

As a widely studied human endothelial cell type, HUVECs (human umbilical vein endothelial cells) are useful for studying the physiological and pathological processes of the vasculature in vitro [66,67]; thus, the antiproliferative activity of **16** was assessed on HUVECs (Table 1). The results showed that **16** also exhibited a strong anti-proliferation effect on HUVECs, with an IC_50_ of 1.48 μM, indicating the potential of anti-angiogenesis activity. Double staining with FDA/PI was then performed to evaluate the cytotoxicity of compound **16** against HUVECs. As shown in Figure 11A, with an increase in the concentration of **16**, the number of vital cells decreased (green), whereas the dead cells stained with red increased, resulting in enhanced cell death relative to the untreated control cells. Further statistical analysis demonstrated that the percentage of dead cells was significantly boosted with increasing concentrations of **16** (*p* < 0.01, Figure 11B), with this effect being more potent than in the case of TET (*p* < 0.01). Specifically, at 5 and 10 μM, very few viable cells were detected in the groups treated with **16**, with viable cell percentages of 1.9% and 0.1%, respectively. 

**Figure 12:**
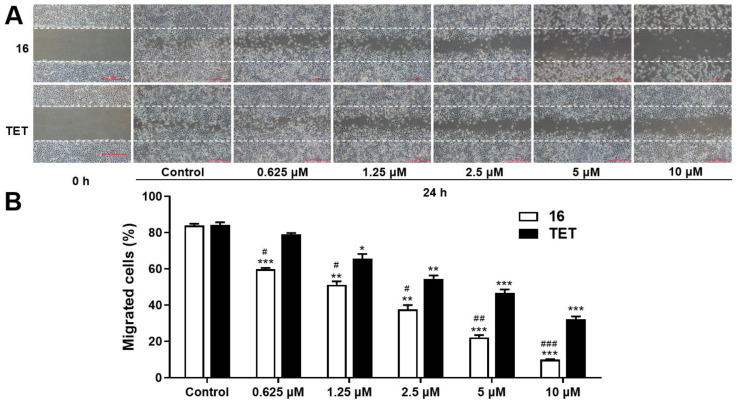
Inhibitory effect of **16** on the migration of HUVECs. (**A**) Photos of HUVECs migration inhibited by **16** (0.625, 1.25, 2.5, 5, and 10 μM). (**B**) Statistical analysis of migrated cells calculated by ImageJ software. *: *p* < 0.05, **: *p* < 0.01, ***: *p* < 0.001, vs. control group; ^#^: *p* < 0.05, ^##^: *p* < 0.01, ^###^: *p* < 0.001, vs. TET at the same concentration.

The inhibitory activity of **16** against the migration of vascular endothelial cell, which is a significant process during angiogenesis, was then investigated. The results demonstrated that, in the control group, the scratched area was almost completely covered after 24 h (Figure 12A), with a migration rate of 85% (Figure 12B). However, compound **16** significantly delayed the recovery of the scratched areas (*p* < 0.05), and few cells covered the blank areas at 10 μM, with a migration rate of 10.0%. Moreover, the migration rate of **16** was much lower than that of TET (*p* < 0.05), suggesting that **16** effectively inhibited the migration of HUVECs. 

**Figure 13:**
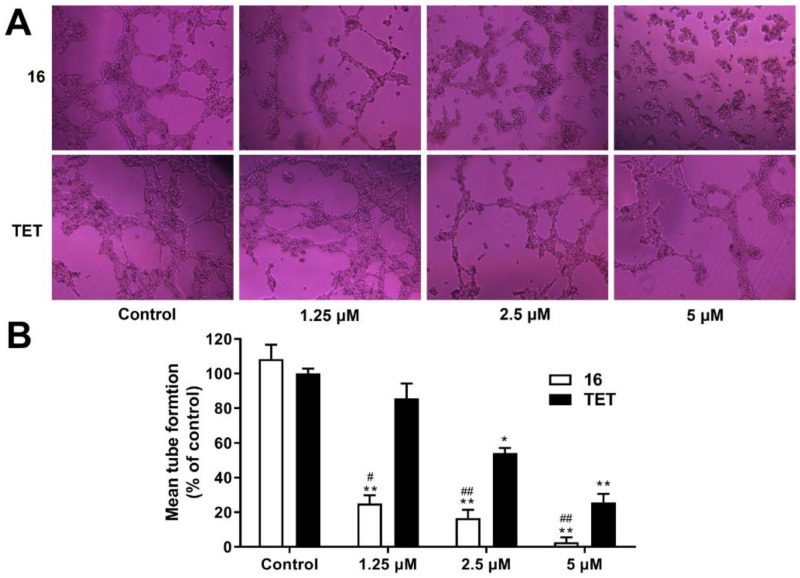
Inhibitory effect of **16** on the tube formation of HUVECs on Matrigel. (**A**) Representative images of the tube formation suppressed by **16** (1.25, 2.5, and 5 μM) in HUVECs (×40). (**B**) Quantitative histogram analysis for the tube-forming capability. *: *p* < 0.05, **: *p* < 0.01 vs. control group; ^#^: *p* < 0.05, ^##^: *p* < 0.01 vs.TET at the same concentration.

Neovascularization is a crucial process during cancer growth, and tube formation assay on HUVEC is commonly used to evaluate the ability of endothelial cells to form capillary-like structure [68]. Thus, this model was then used to analyze the anti-angiogenesis effect of **16**. As illustrated in Figure 13A, integrated tubes on the Matrigel matrix were observed in the control groups, whereas the tubes were fragmentary with the treatment of **16**. The tube length, tube area and number of branch points of endothelial cells were obviously reduced with increasing concentration of **16**. Compared with the control group, the HUVEC tube forming capacity was significantly inhibited by **16** at 5 μM (*p* < 0.01, Figure 13B), with almost no tube structure was observed.

## 3. Materials and Methods

### 3.1. Instruments and Materials 

All the reagents and chemicals were purchased from commercial sources. Bicinchoninic acid (BCA) was purchased from Nanjing Jiancheng Bioengineering Institute (Nanjing, China). Anti-B-cell lymphoma 2 (Bcl-2) antibody, anti-Bcl-2 associated X protein (Bax) antibody, cysteine protease-3 antibody (caspase3), and β-actin antibody were purchased from ProteinTech (Wuhan, China). Anti-Beclin-1 and anti-p62/SQSTM1 (P62) were purchased from Abcam (Cambridge, ENG), and anti-microtubule-associated protein 1 light chain 3 (LC3) was purchased from Cell Signaling Technology (Danvers, MA, USA). 

IR spectra were measured by the Perkin-Elmer Spectrum One FT-IR spectrometer (as KBr pieces; in cm^−1^) (Perkin-Elmer, Boston, MA, USA). ^1^H NMR and ^13^C NMR spectra were recorded on a JEOL spectrometer (400 MHz) using CDCl_3_ or CD_3_OD as solvents with an internal standard of tetramethylsilane. HRMS was recorded on a Thermo Scientific Q Exactive Plus Orbitrap liquid chromatograph triple quadrupole mass spectrometer (LC-MS/MS). All prepared compounds were purified to >96% purity as determined through HPLC (Thermo Scientific Dionex Ultimate 3000, Waltham, MA, USA) analysis using the following methods. A SuperLu C18(2) (particle size = 5 μm, pore size = 4.6 nm, dimensions = 250 mm) column was used, the injection volume was 10 μL, and the mobile phase consisted of methyl alcohol and 0.1% trimethylamine (85:15) with a gradient elution at a flow rate of 1.0 mL/min. Each analysis lasted for 20 min. The detection wavelength was 280 nm. The retention times (R_T,HPLC_) and purity data (%) are listed in the analytical data of each compound. 

### 3.2. Methods of Synthesis 

#### 3.2.1. General Procedure for the Preparation of 14-Nitrotetrandrine (**1**)

The synthesis of compound **1** followed the reported method [69]. Concentrated HNO_3_ (0.6 mL, 9.6 mmol) was slowly added dropwise to a solution of (CH_3_CO)_2_O (1.5 mL, 16.0 mmol) at –10 °C under the protection of a nitrogen atmosphere. Then, 10 min later, the reaction mixture was warmed up to 0 °C and stirred for 20 min, and TET (0.5 g, 0.8 mmol) dissolved in CH_2_Cl_2_ (20 mL) was added dropwise. Upon completion, the mixture was quenched with water (50 mL), extracted with CH_2_Cl_2_ (50 mL × 3), dried over by anhydrous magnesium sulfate, and filtered. The solvent was concentrated under reduced pressure, and the syrup was purified by silica gel chromatography from CH_2_Cl_2_/MeOH (60/1 *v*/*v*) to provide compound **1**, with a yield of 94%. 

#### 3.2.2. General Procedure for the Preparation of 14-Aminotetrandrine (**2**) 

The preparation of compound **2** was performed according to the reported literature [33]. Compound **1** (1.0 g, 1.5 mmol) was added to a mixture of SnCl_2_•2H_2_O (1.70 g, 7.5 mmol) in EtOAc (50 mL), and the reaction was stirred at 80 °C for 4 h. The mixture was then cooled to room temperature, brought to a pH of 8 by adding anhydrous sodium carbonate, and concentrated under reduced pressure to provide the crude product. The residue was purified by silica gel chromatography from CH_2_Cl_2_/MeOH (50/1 *v*/*v*, 0.1% TEA) to afford compound **2**, with a yield of 45%.

#### 3.2.3. General Procedure for the Synthesis of Compounds **3**, **7**, **8**, and **11**

Boc-*L*-amino acid (263 mg, 1.5 mmol) and DCC (162 mg, 0.78 mmol) were added to a solution of **2** (500 mg, 0.78 mmol) in CH_2_Cl_2_ (30 mL) under the protection of nitrogen. The mixture was stirred at room temperature for 3 h, which was monitored by TLC. Upon completion, the mixture was extracted with CH_2_Cl_2_ (15 mL × 3). The combined organic phase was washed with brine (25 mL), dried over anhydrous sodium sulfate, and concentrated under reduced pressure. The crude product was purified using chromatography on silica gel (CH_2_Cl_2_/MeOH, 50/1 *v*/*v*) to provide the pure product. 

*14-N-(Boc-L-glycine)-tetrandrine* (**3**) White solid, 89% yield. IR (KBr) *υ*_max_: 3422, 2936, 1709, 1506, 1450, 1409, 1268, 1221, 1166, 1101, and 879 (cm^−1^). ^1^H NMR (400 MHz, CDCl_3_) *δ* 12.47 (br s, 1H), 7.86 (s, 1H), 7.38 (dd, *J* = 8.0, 2.0 Hz, 1H), 7.24 (dd, *J* = 8.0, 2.4 Hz, 1H), 6.59 (dd, *J* = 8.4, 2.8 Hz, 1H), 6.53 (d, *J* = 7.2 Hz, 2H), 6.33 (s, 1H), 6.12 (dd, *J* = 8.4, 2.0 Hz, 1H), 5.90 (s, 1H), 5.60 (s, 1H), 5.30 (s, 1H), 4.28 (dd, *J* = 11.2, 4.8 Hz, 1H), 3.98–3.89 (m, 6H), 3.84 (d, *J* = 4.4 Hz, 1H), 3.76 (s, 3H), 3.61–3.57 (m, 2H), 3.40 (s, 3H), 3.36–3.31 (m, 1H), 3.13 (s, 3H), 3.04–2.98 (m, 3H), 2.81 (s, 3H), 2.77 (t, *J* = 12.0 Hz, 1 H), 2.51 (s, 3H), 2.42 (d, *J* = 14.8 Hz, 1H), 1.48 (s, 9H). ^13^C NMR (100 MHz, CDCl_3_) *δ* 175.11, 167.03, 156.97, 156.06, 155.91, 152.13, 150.04, 148.94, 148.58, 145.02, 144.91, 137.99, 132.83, 132.41, 130.77, 130.18, 127.38, 125.30, 124.93, 122.36, 121.44, 121.21, 120.88, 120.64, 112.12, 106.44, 106.07, 79.80, 79.19, 63.56, 61.18, 60.15, 56.29, 55.74, 55.59, 44.22, 43.91, 43.45, 43.02, 40.56, 40.01, 39.88, 38.44, 31.57, 30.18, 29.75, 28.48, 22.54, 20.53. HRMS (*m*/*z*): calcd C_45_H_55_N_4_O_9_ for [M+H]^+^, 795.3969; found, 795.3946. R_T,HPLC_ = 7.13 min, purity > 99%. 

*14-N-(Boc-L-proline)-tetrandrine* (**7**) White solid, 87% yield. IR (KBr) *υ*_max_: 3450, 2937, 1691, 1506, 1408, 1223, 1164, 1122, 1008, 881, and 853 (cm^−1^). ^1^H NMR (400 MHz, CDCl_3_) *δ* 11.98 (d, *J* = 14.8 Hz, 1H), 8.01 (s, 1H), 7.29 (dd, *J* = 8.4, 2.4 Hz, 1H), 7.21 (dd, *J* = 8.0, 2.4 Hz,, 1H), 6.58–6.55 (m, 2H), 6.47 (s, 1H), 6.31 (d, *J* = 8.0 Hz, 1H), 6.12 (dd, *J* = 8.4, 2.0 Hz, 1H), 5.88 (d, *J* = 5.2 Hz, 1H), 4.12 (q, *J* = 4.4 Hz, 1H), 4.01–3.98 (m, 1H), 3.94 (s, 3H), 3.82–3.78 (m, 1H), 3.75 (s, 3H), 3.71–3.63 (m, 1H), 3.56–3.52 (m, 1H), 3.47–3.40 (m, 2H), 3.36 (s, 3H), 3.23 (q, *J* = 5.6 Hz, 1H), 3.08 (s, 3H), 3.05–2.96 (m, 2H), 2.91–2.85 (m, 2H), 2.76 (t, *J* = 12 Hz, 1H), 2.70–2.65 (m, 1H), 2.60 (s, 3H), 2.49 (s, 3H), 2.46–2.38 (m, 1H), 2.16–2.09 (m, 2H), 2.03–1.89 (m, 2H), 1.47 (s, 3H), 1.40 (s, 6H). ^13^C NMR (100 MHz, CDCl_3_) *δ* 171.22, 156.27, 154.27, 152.32, 149.58, 148.62, 148.49, 144.70, 144.26, 138.47, 133.97, 132.92, 129.67, 128.71, 127.89, 127.07, 124.94, 124.49, 121.72, 121.50, 121.31, 120.94, 120.63, 112.35, 105.90, 105.80, 79.94, 79.47, 64.24, 61.44, 61.24, 60.74, 60.12, 56.24, 55.82, 55.62, 46.99, 45.12, 43.81, 42.53, 40.97, 40.34, 38.83, 31.78, 30.83, 28.62, 28.43, 24.91, 23.91, 21.03. HRMS (*m*/*z*): calcd C_48_H_59_N_4_O_9_ for [M+H]^+^, 835.4282; found, 835.4261. R_T,HPLC_ = 9.06 min, purity > 98%. 

*14-N-(Boc-L-serine)-tetrandrine* (**8**) White solid, 90% yield. IR (KBr) *υ*_max_: 3425, 2936, 1710, 1505, 1450, 1409, 1220, 1166, 1107, 878, and 834 (cm^−1^). ^1^H NMR (400 MHz, CDCl_3_) *δ* 12.58 (s, 1H), 7.76 (s, 1H), 7.29 (dd, *J* = 8.4, 2.4 Hz, 1H), 7.20 (dd, *J* = 8.0, 2.8 Hz, 1H), 6.59 (s, 1H), 6.55 (dd, *J* = 8.4, 2.4 Hz, 1H), 6.47 (s, 1H), 6.32 (s, 1H), 6.13 (dd, *J* = 8.4, 2.0 Hz, 1H), 5.89–5.87 (m, 2H), 4.35 (d, *J* = 4.4 Hz, 1H), 4.00–3.96 (m, 2H), 3.94 (s, 3H), 3.79 (q, *J* = 5.6 Hz, 1H), 3.75 (s, 3H), 3.67–3.52 (m, 2H), 3.47–3.43 (m, 1H), 3.36 (s, 3H), 3.24–3.17 (m, 2H), 3.09 (s, 3H), 3.03–2.99 (m, 2H), 2.91–2.83 (m, 2H), 2.76 (t, *J* = 11.6 Hz, 6H), 2.59 (s, 3H), 2.52 (s, 3H), 2.48–2.42 (m, 3H), 1.48 (s, 9H). ^13^C NMR (100 MHz, CDCl_3_) *δ* 167.72, 156.64, 155.99, 152.27, 149.50, 148.61, 148.30, 145.47, 144.21, 138.31, 134.26, 133.00, 131.76, 129.72, 128.70, 127.88, 127.23, 125.56, 121.57, 121.15, 121.03, 120.70, 112.33, 105.88, 80.35, 66.21, 64.17, 61.26, 60.12, 57.64, 56.30, 55.79, 55.60, 45.10, 43.12, 42.54, 40.69, 39.93, 38.85, 28.44, 24.89, 20.71. HRMS (*m*/*z*): calcd C_46_H_57_N_4_O_10_ for [M+H]^+^, 825.4075; found, 825.4056. R_T,HPLC_ = 6.33 min, purity > 99%. 

*14-N-(Boc-L-tyrosine)-tetrandrine* (**11**) White solid, 93% yield. IR (KBr) *υ*_max_: 3422, 2936, 1710, 1612, 1506, 1450, 1221, 1166, 1100, 877, and 833 (cm^−1^). ^1^H NMR (400 MHz, CDCl_3_) *δ* 12.21 (br s, 1H), 7.55 (s, 1H), 7.31 (dd, *J* = 8.0, 2.0 Hz, 1H), 7.21 (dd, *J* = 8.0, 2.8 Hz, 1H), 7.12 (d, *J* = 8.4 Hz, 2H), 6.76 (d, *J* = 8.4, Hz, 2H), 6.61 (dd, *J* = 8.4, 2.4 Hz, 1H), 6.55 (s, 1H), 6.47 (s, 1H), 6.31 (s, 1H), 6.11 (dd, *J* = 8.4, 2.0 Hz, 1H), 5.87 (s, 1H), 5.43 (d, *J* = 8.4 Hz, 1H), 4.42 (q, *J* = 6.0 Hz, 1H), 3.93–3.90 (m, 4H), 3.85 (q, *J* = 6.0 Hz, 1H), 3.74 (s, 3H), 3.34 (s, 3H), 3.32–3.28 (m, 1H), 3.21–3.10 (m, 5H), 3.08 (s, 3H), 3.03–2.89 (m, 5H), 2.80–2.73 (m, 2H), 2.61 (s, 3H), 2.47 (dd, *J* = 16.8, 4.0 Hz, 1H), 2.40–2.37 (m, 4H), 1.44 (s, 9H). ^13^C NMR (100 MHz, CDCl_3_) *δ* 169.48, 155.32, 152.18, 149.33, 148.93, 148.21, 144.40, 138.18, 132.98, 130.74, 129.83, 127.95, 127.35, 125.95, 124.45, 121.48, 121.23, 120.88, 115.56, 112.34, 105.92, 100.00, 79.75, 64.14, 61.40, 60.14, 56.33, 55.80, 55.62, 44.75, 42.00, 40.83, 32.00, 31.58, 31.51, 31.32, 30.26, 30.21, 29.78, 29.73, 29.44, 28.46, 22.77, 20.70. HRMS (*m*/*z*): calcd C_52_H_61_N_4_O_10_ for [M+H]^+^, 901.4388; found, 901.4364. R_T,HPLC_ = 7.34 min, purity > 99%. 

#### 3.2.4. General Procedure for the Synthesis of Compounds **12**, **14**–**18**, and **20**

TFA (193 mg, 1.69 mmol) was added to a solution of compound **3** (389 mg, 0.56 mmol) in CH_2_Cl_2_ (5 mL) and stirred for 2 h at room temperature. The mixture was quenched with a saturated aqueous solution of sodium bicarbonate and extracted three times with CH_2_Cl_2_ (20 mL × 3). The combined organic phase was dried over anhydrous sodium sulfate before vacuum suction filtration. The crude product was chromatographed on silica gel (CH_2_Cl_2_/MeOH, 40/1 *v*/*v*, 0.1% TEA) to afford compound **12**. The same procedure was also followed for the synthesis of **14**–**18**, **20**.

*14-N-(L-glycine)-tetrandrine* (**12**) White solid, 86% yield. IR (KBr) *υ*_max_: 3459, 2934, 1609, 1508, 1450, 1207, 1135, 840, 802, and 724 (cm^−1^). ^1^H NMR (400 MHz, CDCl_3_) *δ* 12.17 (s, 1H), 7.91 (s, 1H), 7.30 (dd, *J* = 8.4, 2.0 Hz, 1H), 7.21 (dd, *J* = 8.0, 2.4 Hz, 1H), 6.59–6.57 (m, 2H), 6.47 (s, 1H), 6.31 (s, 1H), 6.13 (dd, *J* = 8.4, 2.0 Hz, 1H), 5.88 (s, 1H), 3.97 (d, *J* = 9.2 Hz, 1H), 3.90 (s, 3H), 3.81 (q, *J* = 5.6 Hz, 1H), 3.75 (s, 3H), 3.58–3.52 (m, 1H), 3.48–3.52 (m, 2H), 3.36 (s, 3H), 3.24 (q, *J* = 6.8 Hz, 1H), 3.09 (s, 3H), 3.03–2.85 (m, 5H), 2.76 (t, *J* = 12 Hz, 1H), 2.71–2.66 (m, 1H), 2.60 (s, 3H), 2.48 (s, 3H), 2.42 (d, *J* = 14.4 Hz, 1H). ^13^C NMR (100 MHz, CD_3_OD) *δ* 158.66, 158.36, 155.49, 152.11, 149.22, 148.33, 148.01, 145.15, 144.02, 138.23, 135.28, 133.30, 132.45, 130.51, 129.28, 128.68, 127.87, 125.52, 121.52, 121.34, 121.24, 120.89, 119.26, 116.28, 113.01, 107.45, 106.87, 63.38, 61.59, 59.68, 56.32, 56.04, 56.00, 42.64, 41.16, 37.04, 35.00, 34.81, 31.66, 30.32, 25.45, 20.66, 19.08. HRMS (*m*/*z*): calcd C_40_H_47_N_4_O_7_ for [M+H]^+^, 695.3445; found, 695.3434. R_T,HPLC_ = 5.14 min, purity > 99%. 

*14-N-(L-valine)-tetrandrine* (**14**) White solid, 87% yield. IR (KBr) *υ*_max_: 3378, 2935, 1613, 1503, 1409, 1350, 1219, 1100, 1069, 877, and 831 (cm^−1^). ^1^H NMR (400 MHz, CDCl_3_) *δ* 11.86 (s, 1H), 7.82 (s, 1H), 7.33 (d, *J* = 2.4 Hz, 1H), 7.31 (d, *J* = 2.0 Hz, 1H), 7.22 (dd, *J* = 8.0 Hz, 1H), 6.61 (d, *J* = 2.4 Hz, 1H), 6.59 (m, 1H), 6.48 (s, 1H), 6.32 (s, 1H), 6.13 (dd, *J* = 8.4 Hz, 1H), 5.90 (s, 1H), 4.00 (d, *J* = 9.2 Hz, 1H), 3.95 (s, 3H), 3.90–3.87 (m, 1H), 3.75 (s, 3H), 3.67–3.61 (m, 1H), 3.54–3.47 (m, 1H), 3.36 (s, 3H), 3.31–3.27 (m, 1H), 3.12 (d, *J* = 6.0 Hz, 1H), 3.11 (s, 3H), 3.03–2.91 (m, 5H), 2.80–2.72 (m, 2H), 2.64 (s, 3H), 2.52 (d, *J* = 4.8 Hz, 1H), 2.48 (s, 3H), 2.44 (s, 1H), 2.40 (s, 1H), 2.06–2.00 (m, 4H), 1.10 (d, *J* =6.8 Hz, 3H), 1.04 (d, *J* = 6.8 Hz, 3H). ^13^C NMR (100 MHz, CD_3_OD) *δ* 173.31, 155.81, 152.15, 149.13, 148.90, 148.03, 145.67, 144.30, 138.15, 134.13, 132.85, 131.31, 129.79, 128.12, 127.42, 127.28, 126.23, 121.24, 121.14, 120.61, 120.37, 120.14, 112.22, 107.43, 105.89, 63.29, 61.70, 59.04, 55.46, 54.80, 54.54, 44.10, 43.29, 41.05, 40.44, 39.36, 36.27, 32.91, 24.40, 20.15, 18.81, 17.02. HRMS (*m*/*z*): calcd C_43_H_53_N_4_O_7_ for [M+H]^+^, 737.3914; found, 737.3877. R_T,HPLC_ = 6.71 min, purity > 99%. 

*14-N-(L-leucine)-tetrandrine* (**15**) White solid, 87% yield. IR (KBr) *υ*_max_: 3385, 2935, 1677, 1611, 1506, 1449, 1408, 1220, 1110, 1070, 878, and 830 (cm^−1^). ^1^H NMR (400 MHz, CD_3_OD) *δ* 7.57 (s, 1H), 7.28 (dd, *J* = 8.4, 2.0 Hz, 1H), 7.12 (dd, *J* = 8.4, 2.8 Hz, 1H), 6.63 (s, 1H), 6.49–6.47 (m, 2H), 6.38 (s, 1H), 6.14 (dd, *J* = 8.4, 2.0 Hz, 1H), 5.82 (s, 1H), 3.99 (q, *J* = 5.2 Hz, 1H), 3.90 (s, 3H), 3.80 (d, *J* = 9.2 Hz, 1H), 3.72–3.69 (m, 4H), 3.64–3.60 (m, 1H), 3.52–3.44 (m, 2H), 3.34 (s, 3H), 3.31–3.30 (m, 1H), 3.23 (q, *J* = 5.6 Hz,1H), 3.00–2.95 (m, 8H), 2.85–2.75 (m, 3H), 2.67 (s, 3H), 2.43 (s, 3H), 2.29 (d, *J* = 14.4 Hz, 1H), 1.87–1.79 (m, 1H), 1.02 (d, *J* = 6.8 Hz, 6H). ^13^C NMR (100 MHz, CD_3_OD) *δ* 155.62, 152.14, 149.13, 149.03, 148.00, 146.10, 144.46, 138.11, 133.87, 132.86, 131.03, 129.92, 127.82, 127.51, 126.68, 121.21, 120.96, 120.86, 120.31, 119.94, 112.29, 108.01, 106.02, 63.22, 61.99, 59.12, 55.57, 54.86, 54.61, 44.28, 43.59, 43.31, 40.94, 40.49, 39.13, 36.04, 24.64, 24.27, 22.10, 21.63, 20.12. HRMS (*m*/*z*): calcd C_44_H_55_N_4_O_7_ for [M+H]^+^, 751.4071; found, 751.4044. R_T,HPLC_ = 8.78 min, purity > 99%. 

*14-N-(L-proline)-tetrandrine* (**16**) White solid, 87% yield. IR (KBr) *υ*_max_: 3447, 2933, 1677, 1628, 1506, 1450, 1407, 1269, 1219, 1108, 1002, 878, and 833 (cm^−1^). ^1^H NMR (400 MHz, CDCl_3_) *δ* 12.28 (br s, 1H), 7.76 (s, 1H), 7.32 (dd, *J* = 8.4, 2.0 Hz, 1H), 7.21 (dd, *J* = 8.0, 2.4 Hz, 1H), 6.58 (dd, *J* = 8.4, 2.4 Hz, 1H), 6.57 (s, 1H), 6.48 (s, 1H), 6.32 (s, 1H), 6.14 (dd, *J* = 8.8, 2.4 Hz, 1H), 5.90 (s, 1H), 4.00–3.97 (m, 2H), 3.94–3.93 (m, 4H), 3.89–3.84 (m, 2H), 3.75 (s, 3H), 3.34 (s, 3H), 3.30–3.25 (m, 2H), 3.18 (d, *J* = 9.6 Hz, 1H), 3.11 (s, 3H), 3.02–2.89 (m, 6H), 2.80–2.72 (m, 2H), 2.65–2.62 (m, 4H), 2.52–2.48 (m, 4H), 2.44–2.40 (m, 2H), 2.30–2.24 (m, 1H), 2.06–1.97 (m, 2H), 1.96–1.88 (m, 1H). ^13^C NMR (100 MHz, CDCl_3_) *δ* 171.58, 155.95, 152.23, 149.34, 148.79, 148.27, 145.36, 144.28, 138.26, 133.88, 132.94, 131.94, 129.83, 128.26, 127.29, 127.25, 125.45, 121.48, 121.19, 121.14, 120.88, 120.75, 112.34, 106.59, 105.88, 64.13, 61.33, 61.26, 60.17, 56.32, 55.81, 55.63, 47.36, 44.92, 43.61, 42.24, 41.05, 39.96, 38.80, 31.68, 29.78, 25.95, 24.64, 20.92. HRMS (*m*/*z*): calcd C_43_H_51_N_4_O_7_ for [M+H]^+^, 735.3758; found, 735.3744. R_T,HPLC_ = 6.78 min, purity > 97%.

*14-N-(L-serine)-tetrandrine* (**17**) White solid, 88% yield. IR (KBr) *υ*_max_: 3377, 2935, 1611, 1505, 1449, 1409, 1268, 1219, 1109, 1069, 875, and 830 (cm^−1^). ^1^H NMR (400 MHz, CD_3_OD) *δ* 7.68 (s, 1H), 7.32 (d, *J* = 6.4 Hz, 1H), 7.12 (dd, *J* = 8.4, 2.8 Hz, 1H), 6.61 (s, 1H), 6.50 (s, 1H), 6.46 (dd, *J* = 8.4, 2.8 Hz, 1H), 6.39 (s, 1H), 6.15 (dd, *J* = 8.4, 2.0 Hz, 1H), 5.85 (s, 1H), 3.92–3.89 (m, 4H), 3.85–3.80 (m, 2H), 3.74 (t, *J* = 5.6 Hz, 1H), 3.71 (s, 3H), 3.47 (t, *J* = 5.6 Hz, 1H), 3.33 (s, 3H), 3.29–3.27 (m, 2H), 3.21 (q, *J* = 6.8 Hz, 1H), 3.01–2.98 (m, 5H), 2.95–2.87 (m, 2H), 2.80–2.74 (m, 2H), 2.60 (s, 3H), 2.43 (s, 3H), 2.28 (d, *J* = 14.4Hz, 1H). ^13^C NMR (100 MHz, CD_3_OD) *δ* 171.99, 155.76, 152.14, 149.12, 148.97, 147.94, 145.77, 144.36, 138.12, 134.22, 132.85, 131.34, 129.84, 128.11, 127.65, 127.30, 126.44, 121.27, 121.23, 120.73, 120.25, 120.07, 112.25, 107.89, 105.91, 64.65, 63.32, 61.80, 59.09, 57.76, 55.40, 54.83, 54.57, 44.13, 43.23, 41.05, 40.31, 39.36, 36.31, 24.38, 20.28. HRMS (*m*/*z*): calcd C_41_H_49_N_4_O_8_ for [M+H]^+^, 725.3550; found, 725.3537. R_T,HPLC_ = 3.95 min, purity > 99%. 

*14-N-(L-threonine)-tetrandrine* (**18**) White solid, 85% yield. IR (KBr) *υ*_max_: 3395, 2934, 1666, 1611, 1506, 1450, 1409, 1268, 1220, 1110, 1070, 1017, 878, and 833 (cm^−1^). ^1^H NMR (400 MHz, CDCl_3_) *δ* 11.99 (s, 1H), 7.86 (s, 1H), 7.31 (dd, *J* = 8.0, 2.0 Hz, 1H), 7.21 (dd, *J* = 8.4, 2.8 Hz, 1H), 6.60–6.57 (m, 2H), 6.47 (s, 1H), 6.32 (s, 1H), 6.13 (dd, *J* = 8.4, 2.0 Hz, 1H), 5.89 (s, 1H), 3.99 (d, *J* = 9.2 Hz, 1H), 3.95 (s, 3H), 3.93 (q, *J* = 5.2 Hz, 3H), 3.75 (s, 3H), 3.65–3.57 (m, 1H), 3.54–3.42 (m, 2H), 3.36 (s, 3H), 3.25 (q, *J* = 5.2 Hz, 1H), 3.10 (s, 3H), 3.05–2.95 (m, 3H), 2.92–2.85 (m, 2H), 2.77 (t, *J* = 11.6 Hz, 1H), 2.71–2.66 (m, 1H), 2.61 (s, 3H), 2.49 (s, 3H), 2.43 (d, *J* = 14.4 Hz,1H), 2.38 (s, 1H), 2.03 (br s, 3H), 1.43 (d, *J* = 6.8 Hz, 3H). ^13^C NMR (100 MHz, CDCl_3_) *δ* 175.02, 156.06, 152.23, 149.51, 148.66, 148.32, 145.00, 144.23, 138.31, 133.98, 132.96, 132.37, 129.74, 128.51, 127.59, 127.12, 125.19, 121.59, 121.39, 121.06, 120.69, 112.28, 106.37, 105.82, 64.17, 61.23, 60.15, 56.25, 55.80, 55.61, 52.07, 45.02, 43.58, 42.44, 40.96, 40.06, 38.80, 24.79, 22.03, 20.79. HRMS (*m*/*z*): calcd C_42_H_51_N_4_O_8_ for [M+H]^+^, 739.3707; found, 739.3691. R_T,HPLC_ = 4.37 min, purity > 98%. 

*14-N-(L-tyrosine)-tetrandrine* (**20**) White solid, 86% yield. IR (KBr) *υ*_max_: 3424, 2936, 1689, 1611, 1507, 1450, 1411, 1269, 1220, 1122, 879 and 833 (cm^−1^). ^1^H NMR (400 MHz, CD_3_OD) *δ* 6.90 (s, 1H), 6.81 (dd, *J* = 6.4, 1.2 Hz, 1H), 6.68–6.65 (m, 3H), 6.39–6.34 (m, 3H), 6.29 (s, 1H), 6.16 (dd, *J* = 6.4, 2.0 Hz, 1H), 6.10 (s, 1H), 6.07 (s, 1H), 5.90 (dd, *J* = 6.8, 1.6 Hz, 1H), 5.63 (s, 1H), 4.29 (q, *J* = 4.4 Hz, 1H)4.08 (s, 3H), 3.96–3.93 (m, 4H), 3.90–3.86 (m, 3H), 3.76–3.70 (m, 1H), 3.62 (s, 3H), 3.60–3.59 (m, 4H), 3.48–3.44 (m, 2H), 3.41–3.39 (m, 2H), 3.37 (s, 3H), 3.34–3.24 (m, 6H), 3.18 (s, 3H), 2.88 (dd, *J* = 8.8, 3.6 Hz,1H), 2.77–2.73 (m, 4H). ^13^C NMR (100 MHz, CD_3_OD) *δ* 157.75, 157.14, 153.40, 150.92, 150.12, 149.22, 147.47, 146.01, 139.30, 134.24, 134.19, 132.04, 131.61, 131.31, 128.96, 128.33, 128.21, 128.09, 126.21, 122.43, 122.27, 122.14, 121.83, 121.00, 119.64, 116.73, 116.62, 113.67, 109.39, 107.41, 101.31, 64.69, 63.23, 60.44, 56.89, 56.19, 55.98, 45.77, 44.50, 41.89, 41.70, 24.93, 21.47. HRMS (*m*/*z*): calcd C_47_H_53_N_4_O_8_ for [M+H]^+^, 801.3863; found, 801.3850. R_T,HPLC_ = 4.14 min, purity > 99%. compounds **4**–**6**, **9**, **10**, **13**, and **19** were obtained according to the reported method, with a purity > 96% [70].

### 3.3. Cell Lines and Culture Conditions

Human lung cancer cell (A549,), human umbilical vein endothelial cells (HUVECs,), human colon cancer cell lines (HCT-15), human breast cancer cells (MCF-7), human liver cancer cells (HepG2), human pancreatic cancer cells (BxPC-3), and human hepatic cell line (L-02) were obtained from the Sichuan University. A549, HUVECs, and L-02 were cultured in DMEM medium (high glucose, HyClone, GE Healthcare, Sydney, Australia). Human colon cancer cell lines HCT-15, human pancreatic cancer cells HCT-15 and BxPC-3 were cultured in RPMI-1640. Human breast cancer cells MCF-7 and human liver cancer cells HepG2 were cultured in MEM medium. All the mediums, including 10% fetal bovine serum (FBS, HyClone, GE Healthcare, Sydney, Australia) and 1% ampicillin/streptomycin, were used to culture the above cells at 37 °C in a humidified atmosphere (5% CO_2_ and 95% air).

### 3.4. MTT Assay

The antiproliferative activity of the title compounds was determined using the MTT (3-(4,5-dimethylthiazol-2-yl)-2,5-diphenyltetrazolium bromide, Sigma-Aldrich, Saint Louis, MO, USA) assay on different cells. Cells lines were seeded into 96-well plates at a density of 2000 to 6000 cells/well. DMSO was used to dissolve the test compounds, and TET was used as a positive control. After 24 h, the test compounds were added at different concentrations (0.625, 1.25, 2.5, 5, 10, and 20 μM) and incubated at 37 °C for 72 h. MTT was added to each well at a final concentration of 0.5 mg/mL and incubated at 37 °C for 4 h. Then, 150 μL of DMSO was added to each well to dissolve the formazan crystals. The absorbance was measured at 570 nm by a microplate reader (ELX800UV, Bio-Tek, Windoski, VT, USA). All measurements were repeated at least three times under the same conditions. Graphpad Prism 8.0 was used to calculate the IC_50_ values.

### 3.5. Aqueous Solubility Determination

The water-solubility of the title compounds was measured according to the reported procedure by reversed-phase HPLC (column: Superlu C 18, 250 × 4.6 mm, 5 μM; isocratic elution with a mobile phase of 85% CH_3_OH/15% H_2_O; injection volume, 20 μL; flow rate: 1.0 mL/min; column temperature, 30 °C; UV detection at the wavelength of the maximum absorbance of each compound) [51]. Briefly, each tested compound (10 mg) was dissolved in 10 mL of CH_3_OH, and the standard solution (0.5 mL) was used to determine the wavelength of maximum absorbance by HPLC. The solution was diluted as necessary, and the solubility was determined by linear regression with R^2^ > 0.999. Each tested compound (5 mg) was ultrasound dissolved in 3 mL of pure water for 1 h at room temperature, and the mixture was then concentrated at 3000 rpm for 15 min. The saturated supernatants were determined by HPLC at the wavelength of the maximum absorption, and the absorbances were obtained. The solubility was calculated according to the standard curve.

### 3.6. Colony Formation Assay

HCT-15 cells were diluted in 2 mL of culture medium and plated in six-well plates at 37 °C in 5% CO_2_. After an overnight incubation, compound **16** (0.625, 1.25, 2.5, 5, and 10 μM) was added, and the cells were cultured for 10 d. Afterwards, the medium was replaced. The cells were washed by cold PBS and fixed with 4% paraformaldehyde. Finally, the cell colonies were stained with a 1% crystal violet solution for 20 min. The plates were recorded, and the colonies were counted digitally using ImageJ software with customized macros. 

### 3.7. Wound Healing Assays

HCT-15 cells or HUVECs were seeded in six-well culture plates and allowed to grow to 80–90% confluence. Subsequently, a cell-free line was manually created by scratching the confluent cell monolayers with a 200 μL sterile pipette tip, and the detached cells were washed with PBS. The cells were then incubated with 10% FBS and different concentrations of **16** (0.625, 1.25, 2.5, 5, and 10 μM). Then, 24 h later, images of the same location were obtained using a microscope (Nikon ECLIPSE TE2000-U, Tokyo, Japan). Each experiment was carried out at least three times.

### 3.8. Invasion Assay

The cell motility inhibitory effect of **16** on HCT-15 cells was evaluated by invasion assay. Briefly, cells were harvested and resuspended in serum-free medium that contained 0.625, 1.25, 2.5, 5, and 10 μM of **16**, and were seeded into the upper wells of a transwell chamber (Millicell, 8 mm pore size, 12-mm diameter Millipore) coated with 50 μL of Matrigel (1:3 dilution in serum-free medium, Corning/BD Biosciences) at a density of 2 × 10^5^ cells/mL. Meanwhile, 600 μL medium containing 10% FBS and corresponding samples were added into the lower chambers. Then, 20 h later, the invading cells were fixed with 4% paraformaldehyde and stained with 0.2% crystal violet for 30 min. Afterwards, the chambers were washed twice with PBS and left to dry. Cells which had migrated to the lower chamber of the transwell were photographed using a digital camera with an inverted microscope (Nikon ECLIPSE TE2000-U, Tokyo, Japan), and the crystal violet positive cells were counted.

### 3.9. F-Actin Phalloidin Staining

The effect of compound **16** on the morphology of HCT-15 cells was evaluated by F-actin staining. Briefly, the cancer cells were seeded in 6-well plates with glass bottoms overnight at 37 °C and were incubated with 0.625, 1.25, 2.5, 5, 10 μM of **16** for 24 h. Then, the medium was removed, and the cells were fixed in 4% paraformaldehyde for 5 min and washed thrice with PBS before staining. A solution (200 μL) of 100 nM in MeOH FITC-phalloidin (Molecular Probes, Eugene, OR, USA) containing 1% BSA was added and treated for 30 min in total darkness. Afterwards, the cells were washed thrice with PBS and stained with 200 μL of DAPI (10 mg/mL, Solarbio Science & Technology,Beijing, China) for 5 min at 37 °C. Photos were acquired by a Nikon ECLIPSE TE2000-U inverted epifluorescent microscope using appropriate filters.

### 3.10. Live/Dead Cell Analysis

HCT-15 or HUVEC cells were cultured in six-well plates for 24 h. The cells were washed with PBS and incubated with medium containing 0.625, 1.25, 2.5, 5, and 10 μM of **16** for 48 h. Afterwards, the medium was replaced, and the cells were washed twice with PBS, resuspended in the solution containing 100 μL FDA (0.02 mg/mL, Sigma, St. Louis, MO, USA) and 30 μL PI (0.02 mg/mL, Sigma, St. Louis, MO, USA), and treated in the dark at room temperature for 10 min. The cells were imaged with a fluorescence microscope (Nikon ECLIPSE TE2000-U, Tokyo, Japan).

### 3.11. Hoechst 33258 Staining

HCT-15 cells were cultured in six-well plates for 24 h. Then, the cells were incubated with medium containing different concentrations of **16** (0.625, 1.25, 2.5, 5, 10 μM) for 48 h. After incubation, the medium was removed, and the cells were washed with PBS, fixed with 4% paraformaldehyde for 30 min, stained in 200 μL of buffer Hoechst 33258 (10 mg/mL, Sigma, St. Louis, MO, USA), and incubated in the dark at room temperature for 10 min. The cells were then imaged using a fluorescence microscope.

### 3.12. Flow Cytometry Analysis

HCT-15 cells were cultured in complete medium in six-well plates for 24 h, and 1.5. 2.5, 5 μM of **16** was added in triplicate for 48 h incubation. The cells were digested with trypsin without EDTA, washed twice with cold PBS, and resuspended in 500 μL binding buffer. The cell suspension was stained successively with 5 μL Annexin V-FITC and 5 μL PI (Keygen Biotech, Nanjing, China), and cultured for 15 min and 5 min in total dark, respectively. Afterwards, the percentages of apoptotic cells were determined by a flow cytometry. Data were analyzed by FlowJo software (Version 10, FlowJo, LLC, Ashland, OR, USA).

### 3.13. Cell Cycle Analysis

HCT-15 cells were cultured in six-well plates overnight at 37 °C. The cells were treated with 1.25, 2.5, 5 μM of **16** or DMSO and incubated for 24 h. Afterwards, the cells were harvested, and 70% ice cold ethanol was used to fix cells at 4 °C. Subsequently, the cells were centrifuged to remove the fixative solution, washed twice with PBS, and exposed to 500 μL of PI/RNase working solution (Keygen Biotech, Nanjing, China) at 4 °C for 30 min in the dark. The cell cycle distribution was analyzed by flow cytometry.

### 3.14. Western Blot

The protein levels in HCT-15 cells were evaluated via Western blot analysis. The cells were cultured in dishes (10^7^ cells per dish) and incubated overnight at 37 °C, 0.625, 1.25, 2.5, and 5 μM of **16** was added in triplicate after 24 h of incubation. Then, the cells were harvested and lysed in Laemmli buffer (glycerol, 1M TRIS pH 6.8, 1% SDS, MQ H_2_O and 20% β-mercaptoethanol). The samples were boiled for 5 min at 95 °C, and the protein content was determined using a BCA kit. Equal amounts of 30 μg of sample were placed on a Tris/HCl gel and separated by SDS-PAGE electrophoresis. Then, the samples were transferred to a PVDF membrane (Milibo, Co., Ltd., Billyka, MA, USA) and blocked with 5% skimmed milk powder for 2 h. Diluted primary antibodies Bcl-2 (1:3000), Bax (1:10,000), caspase-3 (l:2000), p62 (1:1000), Beclin1 (1:1000), LC3 (1:2000), and β-actin (1:1000) were incubated overnight at 4 °C, followed by a horseradish peroxidase-conjugated goat anti-rabbit IgG secondary antibody (ProteinTech, China) for 2 h at room temperature. Then, immunoreactivity was developed with diaminobenzidine solution, and scanned and recorded by a gel imaging system (G:BOX Chemi XL1.4, Frederick, MD, USA).

### 3.15. Molecular Docking

PKC-α was chosen as the target receptor, and the 3D structure of the receptor was obtained from the Protein Data Bank (PDB ID: 4RA4). The ligand (**16**) was prepared for docking as described in our previous work [71]. Docking studies were performed with the AutoDock (4.2) program suite. The 3D affinity map was a cube with 60 Å × 60 Å × 60 Å grid points separated by 0.375 Å, and the docking parameters were identical to our previous work [71]. The resulting docked orientations within a root mean square deviation of 2 Å were clustered together.

### 3.16. Tube Formation Assay

Briefly, a 96-well plate was pre-coated with 50 μL Matrigel per well and incubated at 37 °C for 30 min. Then, 100 μL of HUVECs suspension (2 × 10^4^) were added to each well. The cells were cultured with 1.25, 2.5, and 5 μM of 16 for 8 h. Subsequently, the tube formation was observed, randomly selected, and recorded by an inverted microscope.

### 3.17. Statistical Analysis

The experimental results were collected from at least three independent experiments and expressed as mean ± SEM. Data were tested for normal distribution (Kolmogorov–Smirnoff test) and subsequently analyzed with one-way ANOVA and *t*-test using GraphPad Prism 8.0, with *p* < 0.05 indicating a significant difference. 

## 4. Conclusions

In summary, a group of 14-*N*-amino acid-substituted derivatives of TET were designed and synthesized, with improved aqueous solubility and anticancer activity compared to TET. Among them, compound **16** exhibited an outstanding antiproliferative activity against HCT-15 cells, with an IC_50_ of 0.57 μM. Moreover, the aqueous solubility of **16** was markedly improved to higher than 5-fold over TET. Compound **16** significantly inhibited the colony formation, migration, and invasion of HCT-15 cells in a concentration-dependent manner. The flow cytometry assay demonstrated that **16** induced the death of HCT-15 cells; however, no significant effect on the cell cycle redistribution was observed for this compound. Further Western blot studies revealed that **16** induced the death of cancer cells through autophagy rather than apoptosis. Compound **16** markedly inhibited the content of P62 and enhanced the Beclin-1 level and the ratio of LC3-II to LC3-I. In addition, the proliferation, migration, and tube formation of HUVECs were significantly inhibited by **16**, suggesting strong anti-angiogenesis activity. Thus, all these results indicated the potential of **16** as a promising anticancer candidate for further preclinical studies.

## Figures and Tables

**Table 1:** Antiproliferative activities of the target compounds against different human cell lines.

Compd.	Antiproliferative Activity (IC_50_/μM) *^a^*						
	A549	HCT-15	HepG2	BXPC-3	MCF-7	HUVEC	L-02
**3**	2.15 ± 0.17	2.42 ± 0.06	1.94 ± 0.27	2.55 ± 0.03	2.91 ± 0.17	1.68 ± 0.21	8.59 ± 0.13
**4**	7.44 ± 1.11	3.28 ± 0.32	3.90 ± 0.62	4.74 ± 0.37	5.78 ± 0.35	4.01 ± 0.41	>20
**5**	7.24 ± 0.41	4.31 ± 0.09	3.54 ± 0.30	4.63 ± 0.09	7.40 ± 0.31	3.04 ± 0.34	>20
**6**	5.54 ± 0.16	3.89 ± 0.47	3.11 ± 0.68	6.39 ± 0.20	4.99 ± 0.66	2.09 ± 0.19	13.58 ± 0.13
**7**	6.40 ± 0.36	1.15 ± 0.17	3.50 ± 1.37	3.10 ± 0.26	5.71 ± 0.29	3.49 ± 0.31	17.54 ± 0.21
**8**	7.45 ± 0.32	5.87 ± 0.30	3.76 ± 1.34	3.55 ± 0.32	5.84 ± 0.25	3.96 ± 0.36	>20
**9**	12.28 ± 0.61	8.57 ± 0.57	6.97 ± 0.34	13.07 ± 0.37	11.29 ± 0.55	4.91 ± 1.05	>20
**10**	1.96 ± 0.24	0.91 ± 0.05	1.54 ± 0.02	2.31 ± 0.08	2.59 ± 0.11	1.80 ± 0.02	15.54 ± 0.11
**11**	6.26 ± 0.41	3.56 ± 0.27	2.81 ± 0.35	3.58 ± 0.15	5.03 ± 0.04	2.16 ± 0.32	11.79 ± 0.61
**12**	4.94 ± 0.45	3.54 ± 0.36	3.19 ± 0.76	3.98 ± 0.16	6.91 ± 0.66	3.19 ± 0.61	>20
**13**	8.12 ± 0.43	4.69 ± 0.47	3.27 ± 1.00	6.95 ± 0.57	11.06 ± 0.26	2.20 ± 0.37	>20
**14**	6.11 ± 0.91	4.02 ± 0.15	3.26 ± 0.05	4.95 ± 0.29	7.96 ± 1.08	2.48 ± 0.15	14.49 ± 0.37
**15**	5.92 ± 0.73	2.71 ± 0.22	3.66 ± 0.82	4.55 ± 0.27	6.74 ± 0.16	3.09 ± 0.36	12.08 ± 0.04
**16**	1.19 ± 0.01	0.57 ± 0.03	1.05 ± 0.03	1.12 ± 0.10	1.46 ± 0.13	1.48 ± 0.01	>20
**17**	7.44 ± 1.02	3.94 ± 0.37	3.13 ± 0.70	3.67 ± 0.19	7.53 ± 0.11	2.88 ± 0.26	>20
**18**	6.81 ± 1.10	5.28 ± 0.37	3.57 ± 0.67	5.60 ± 0.38	10.24 ± 0.40	3.25 ± 0.69	>20
**19**	2.67 ± 0.14	3.39 ± 0.07	1.71 ± 0.46	1.75 ± 0.13	3.31 ± 0.01	1.92 ± 0.17	9.02 ± 0.26
**20**	4.12 ± 0.27	3.33 ± 0.08	1.44 ± 0.18	2.36 ± 0.15	3.70 ± 0.91	1.91 ± 0.09	10.03 ± 1.28
**TET**	6.39 ± 0.83	6.12 ± 0.51	3.25 ± 0.11	7.60 ± 0.16	3.90 ± 0.47	3.62 ± 0.02	16.02 ± 1.18

*^a^* IC_50_ values are average for three independent experiments.

## Data Availability

No applicable.

## Supplementary Materials

The following supporting information can be downloaded at: https://www.mdpi.com/article/10.3390/molecules27134040/s1. Figure S1. Morphology of HCT-15 cells after adding different concentrations of 16, Figure S2. Cell cycle analysis of HCT-15 cells treated with 16, and ^1^H and ^13^C NMR spectra of compounds **3**, **7**, **8**, **11**, **12**, **14**–**18**, and **20**.

## Sample Availability

Samples of the compounds **1**–**20** are available from the authors.
