# Supplementary material for: Design, Synthesis, and Biological Evaluation of N14-Amino Acid-Substituted Tetrandrine Derivatives as Potential Antitumor Agents against Human Colorectal Cancer
Source: Molecules. 2022 Jun 23;27(13):4040. doi: 10.3390/molecules27134040 (PMC9268013; doi:10.3390/molecules27134040)
Supplement: Supplementary file 1 [file molecules-27-04040-s001.zip › molecules-1742238-supplementary.pdf]

Supporting Information

Design, Synthesis, and Biological Evaluation of N14-Amino Acid-Substituted Tetrandrine Derivatives as Potential Antitumor Agents against Human Colorectal Cancer

Yu-Chan Wang, Rong-Hong Zhang, Sheng-Cao Hu, Hong Zhang, Dan Yang, Wen-Li Zhang, Yong-Long Zhao, Dong-Bing Cui, Yong-Jun Li, Wei-Dong Pan, Shang-Gao Liao, Meng Zhou

Table of Content

1. Supporting Figures

Figure S1……………………………………………………………………………………2

Figure S2…………………………………………………………………………...…2

2. ^{1}H and ^{13}C NMR spectra of compounds…………………………………………….…3

3. IR spectra of compounds……………………………………………….………………14

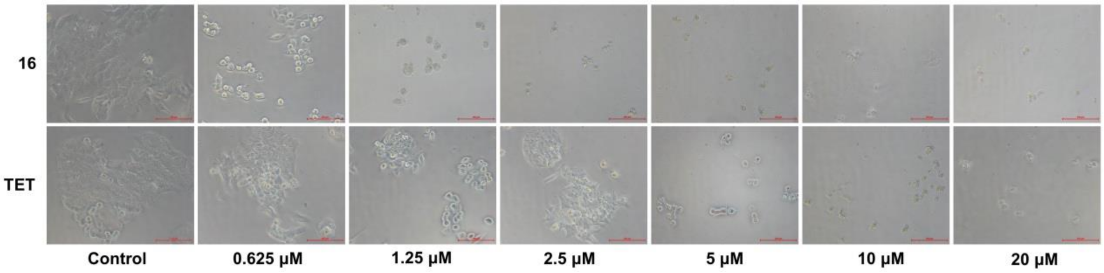

Figure S1. Morphology of HCT-15 cells after adding different concentrations of **16**, and tetrandrine (TET).

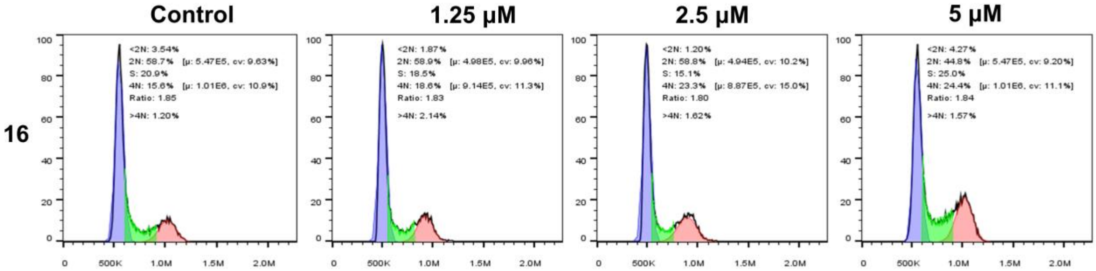

Figure S2. Cell cycle analysis of HCT-15 cells treated with **16** (1.25, 2.5, 5 μM) for 24 h by flow cytometry assay.

^{1}H and ^{13}C NMR spectra of compounds

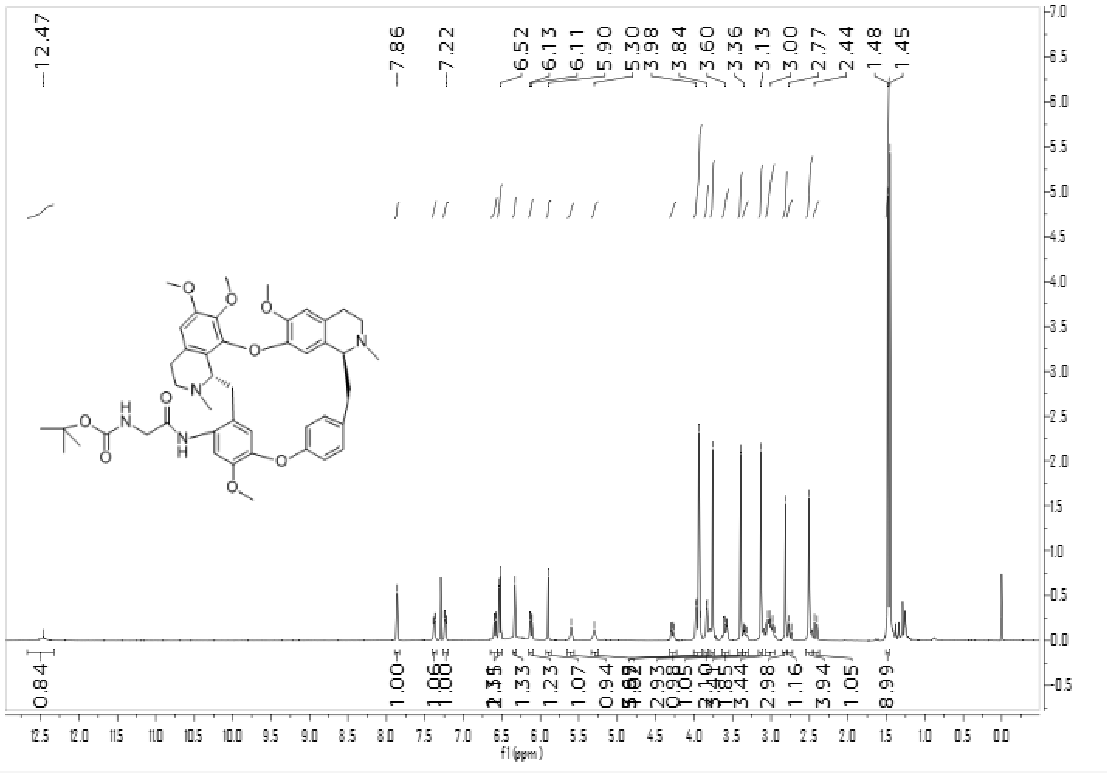

^{1}H NMR spectrum of **3**

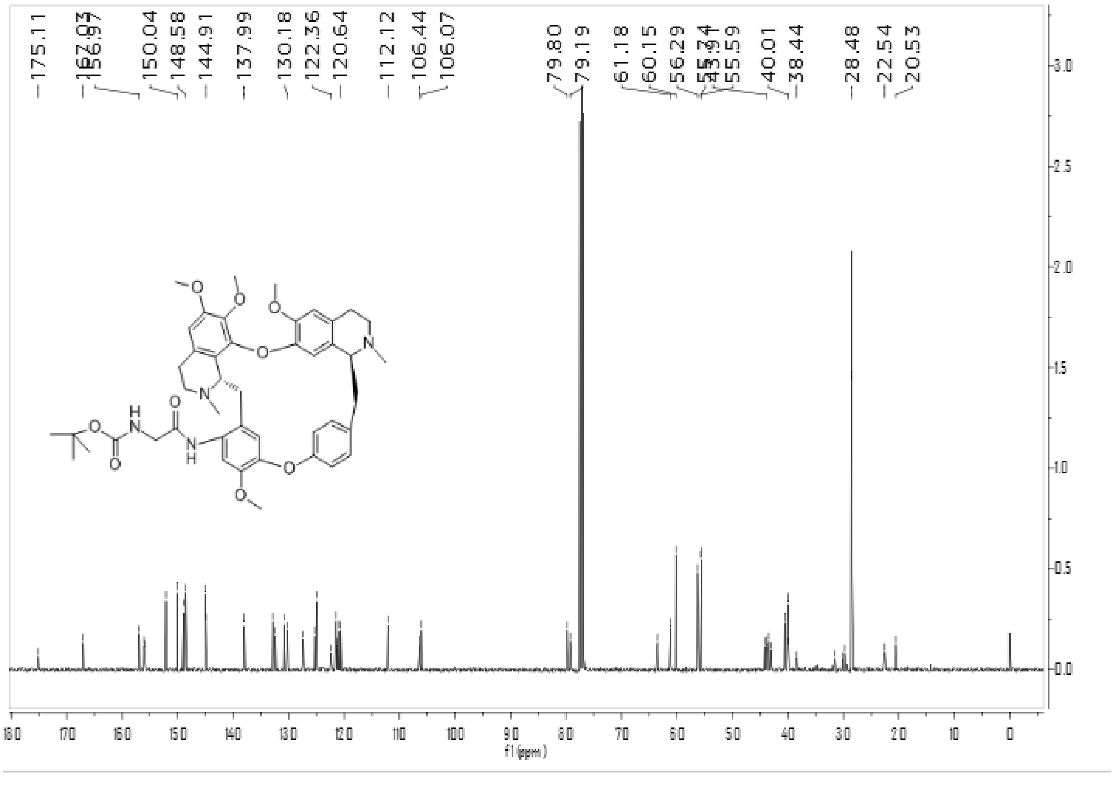

^{13}C NMR spectrum of **3**

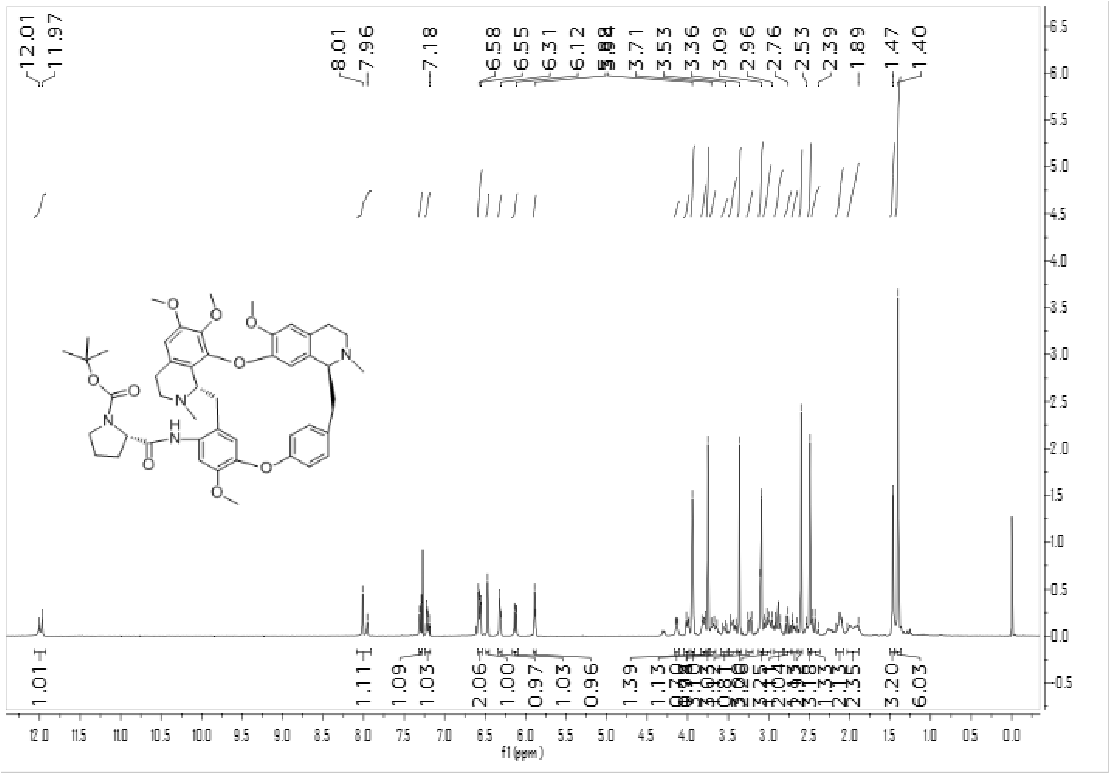

^{1}H NMR spectrum of **7**

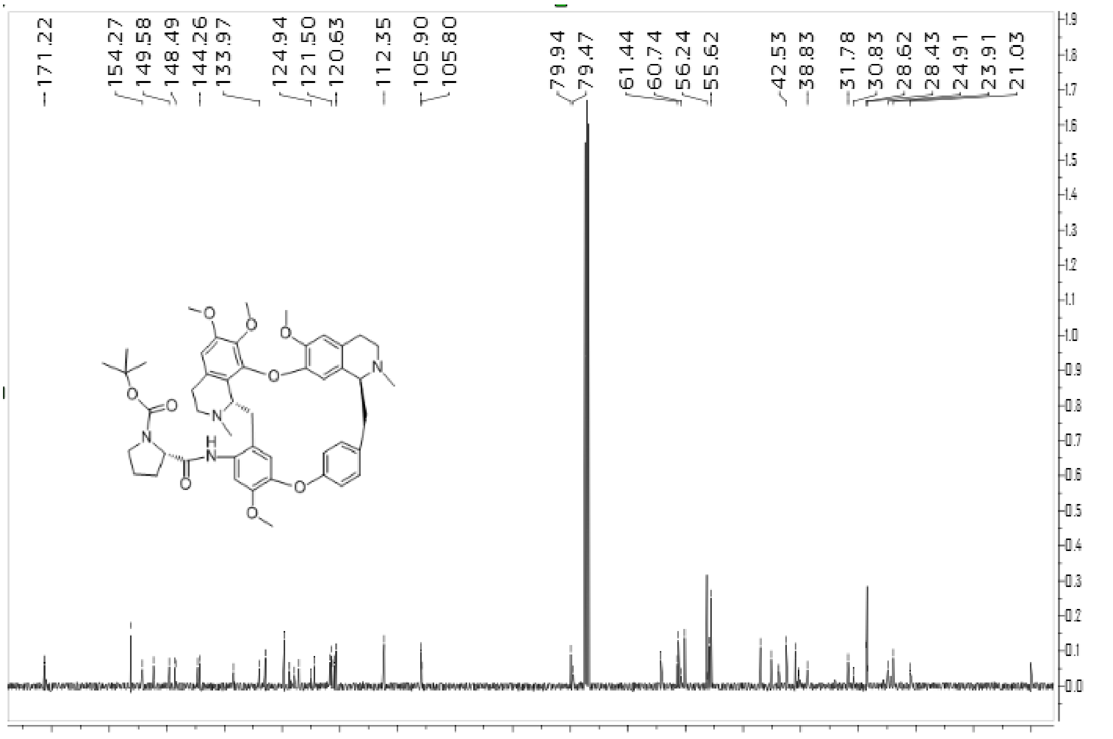

^{13}C NMR spectrum of **7**

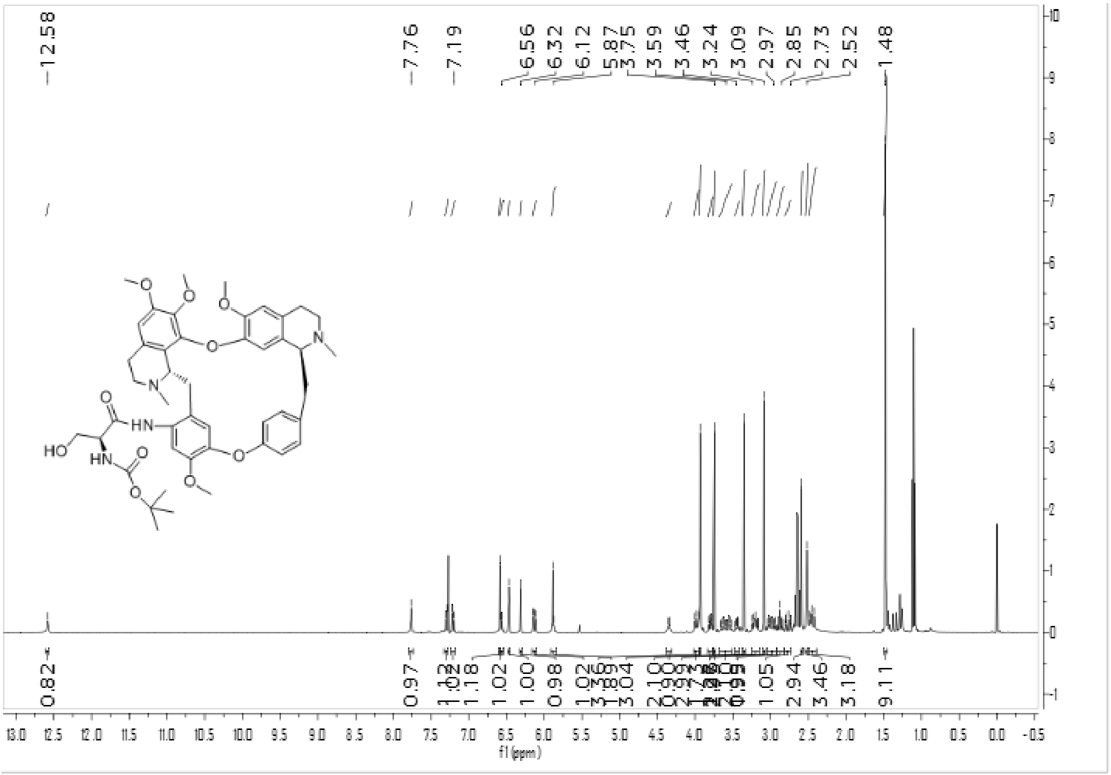

^{1}H NMR spectrum of **8**

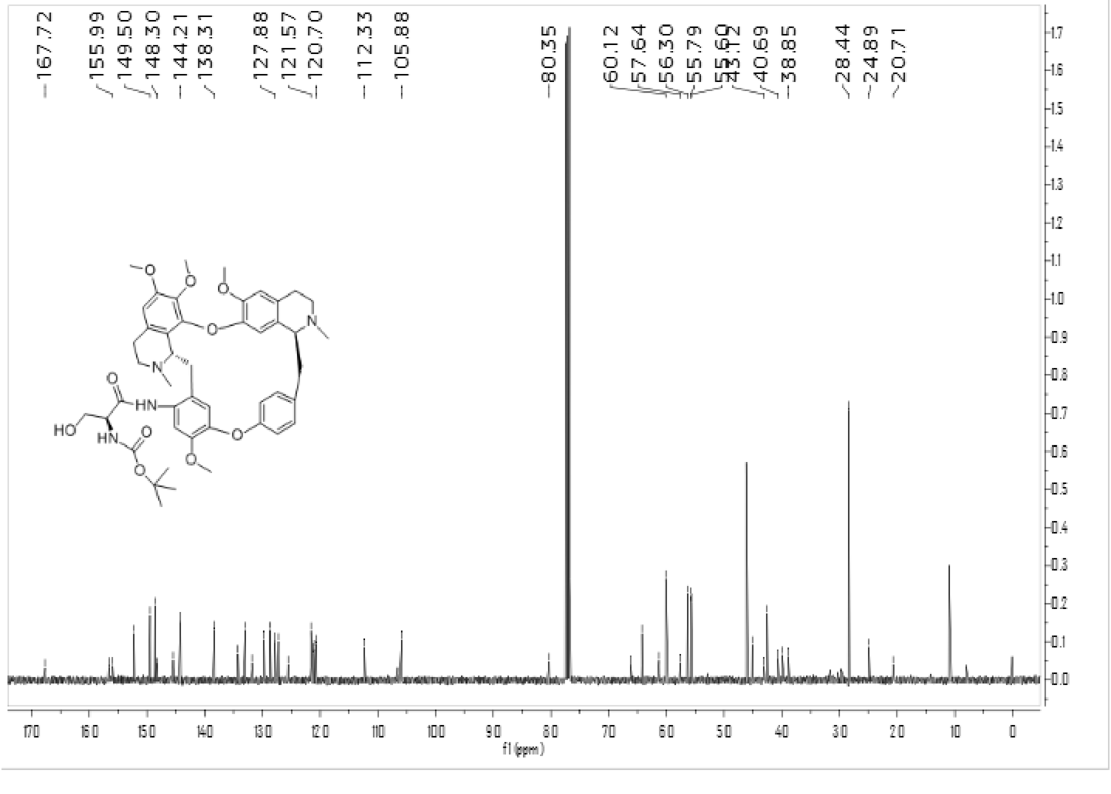

^{13}C NMR spectrum of **8**

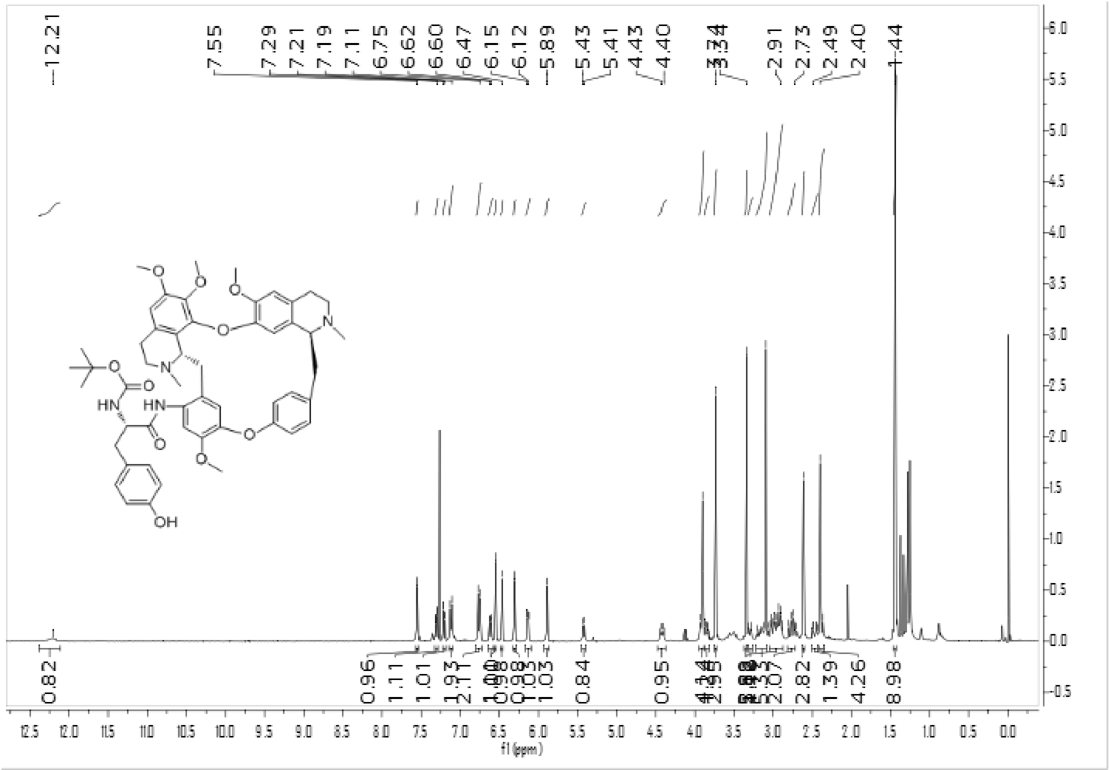

^{1}H NMR spectrum of **11**

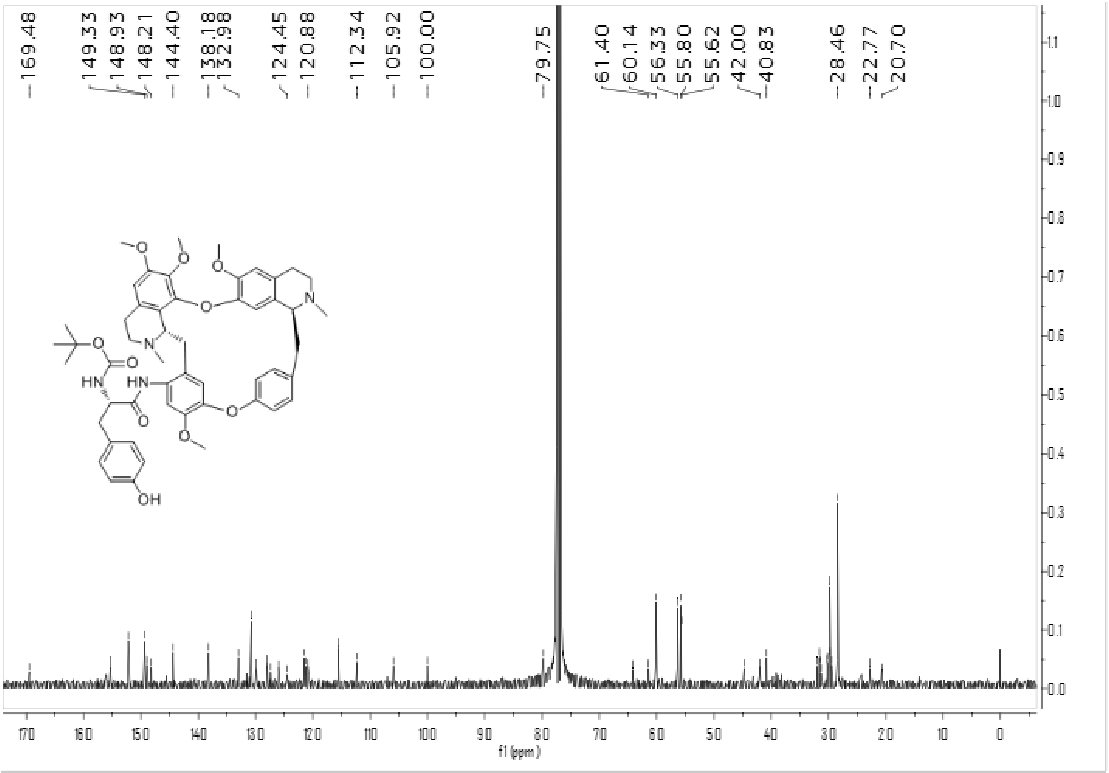

^{13}C NMR spectrum of **11**

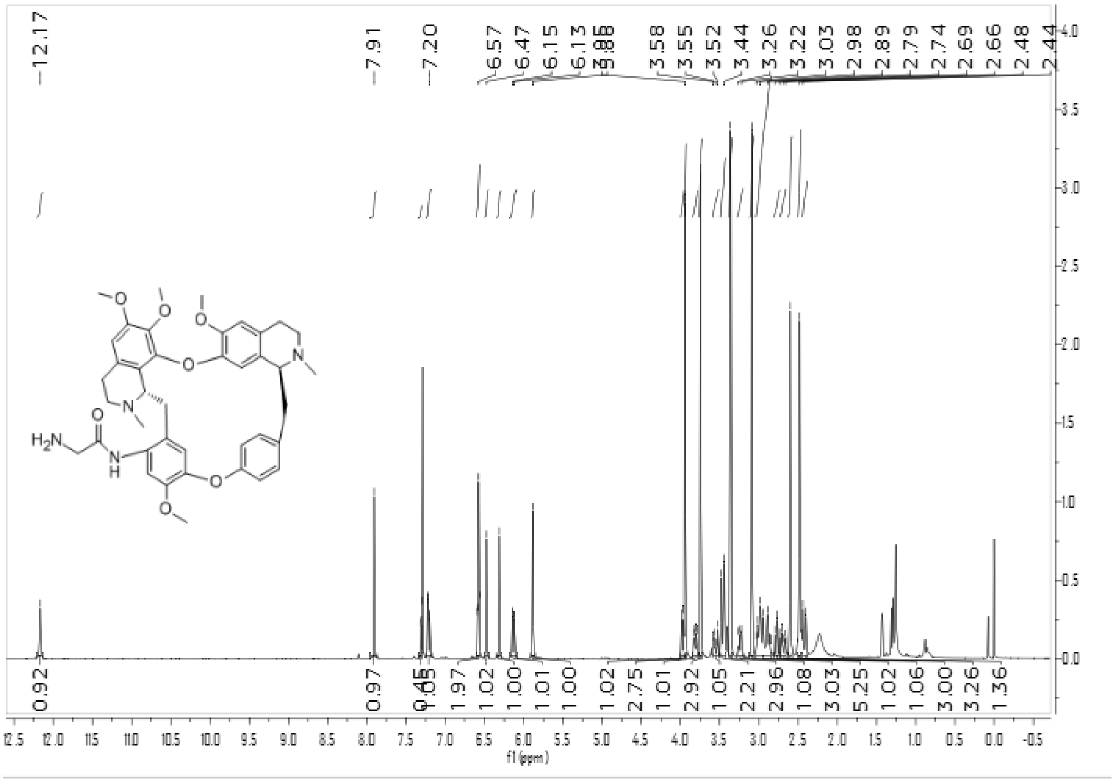

^{1}H NMR spectrum of **12**

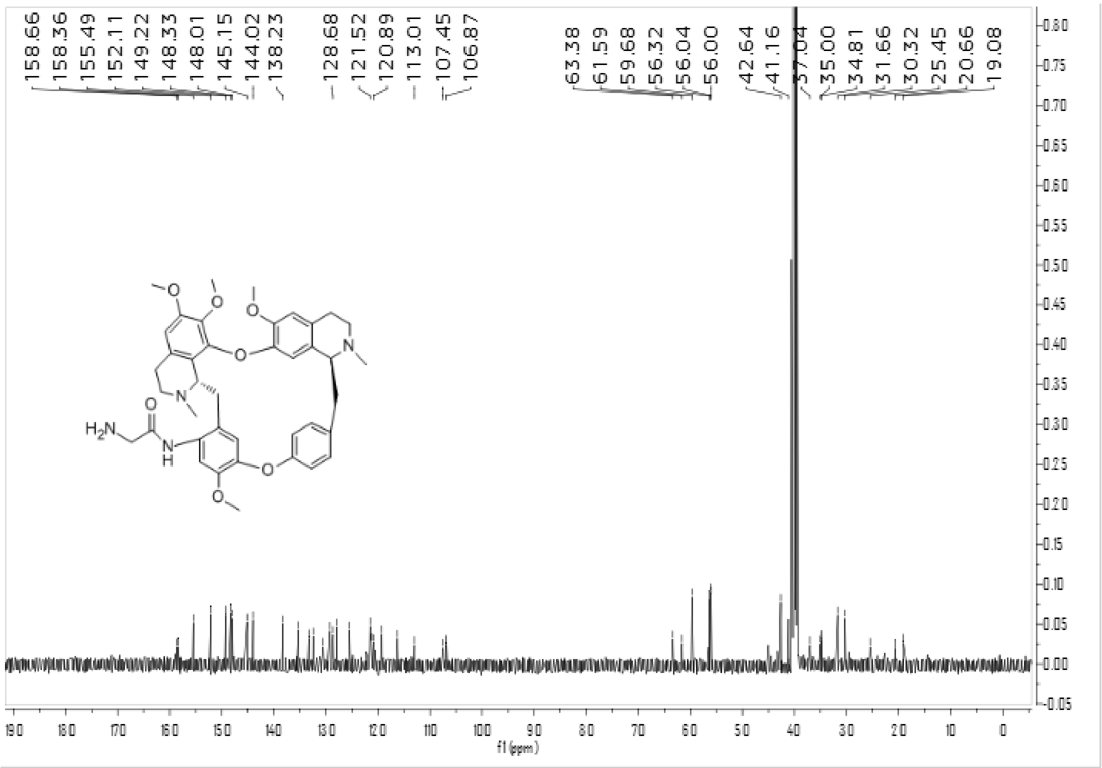

^{13}C NMR spectrum of **12**

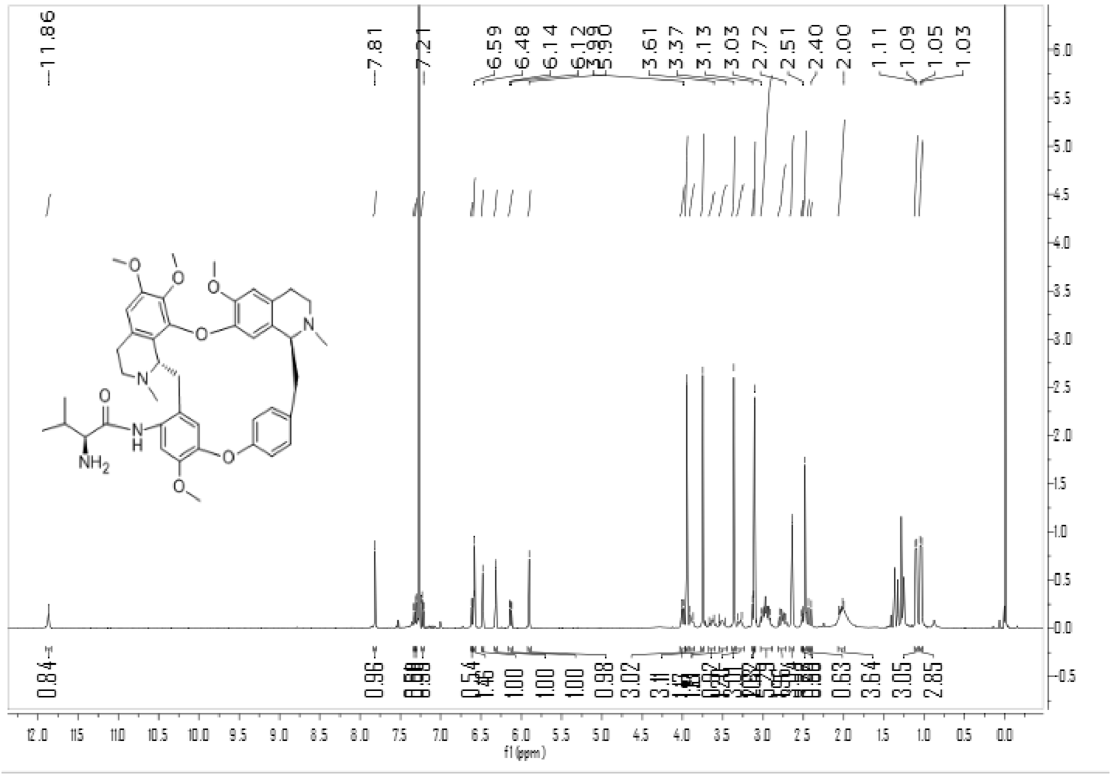

^{1}H NMR spectrum of **14**

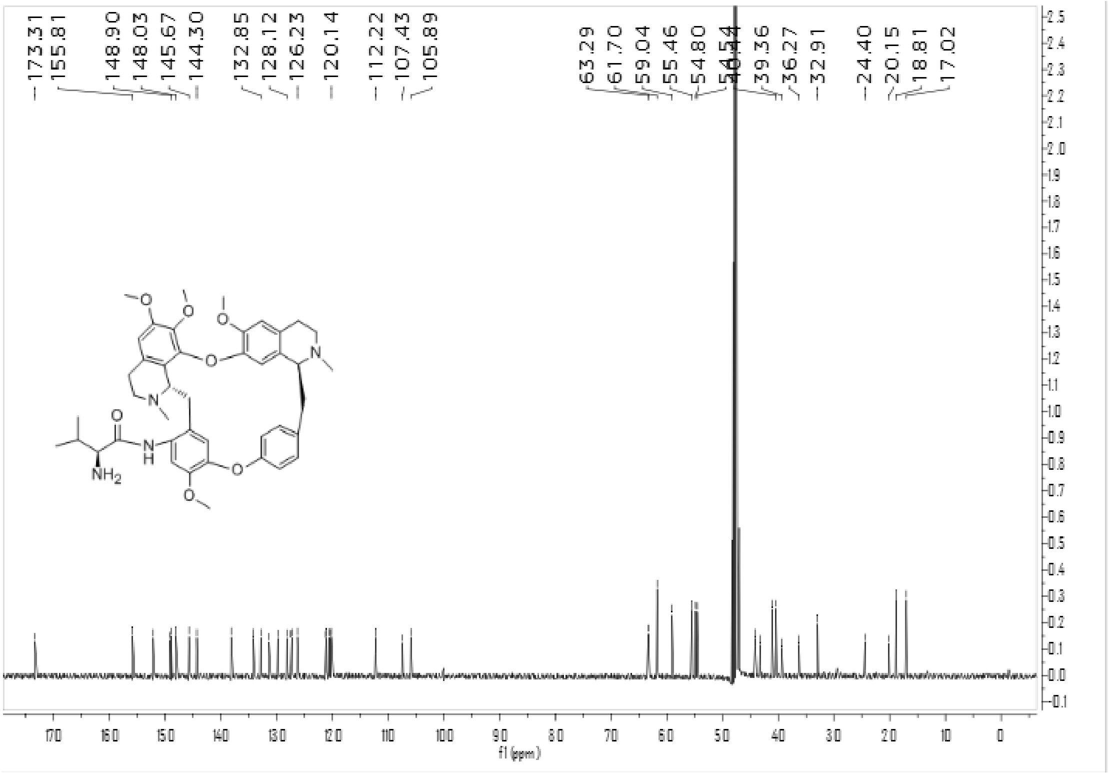

^{13}C NMR spectrum of **14**

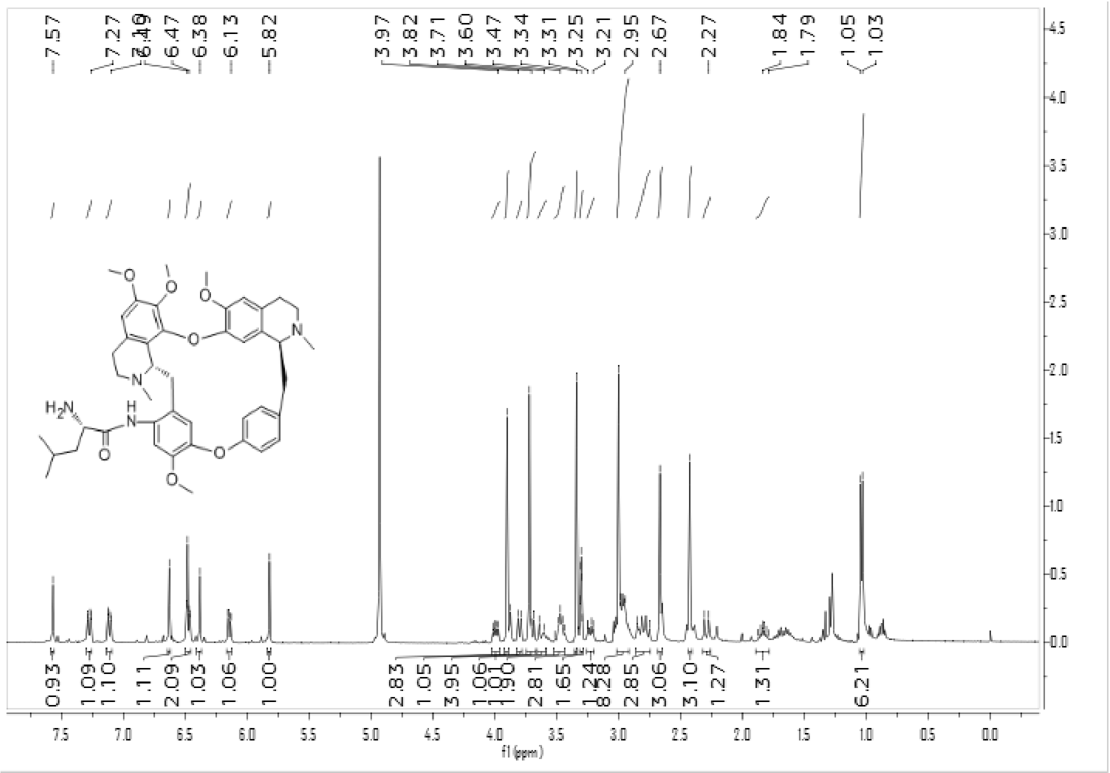

^{1}H NMR spectrum of **15**

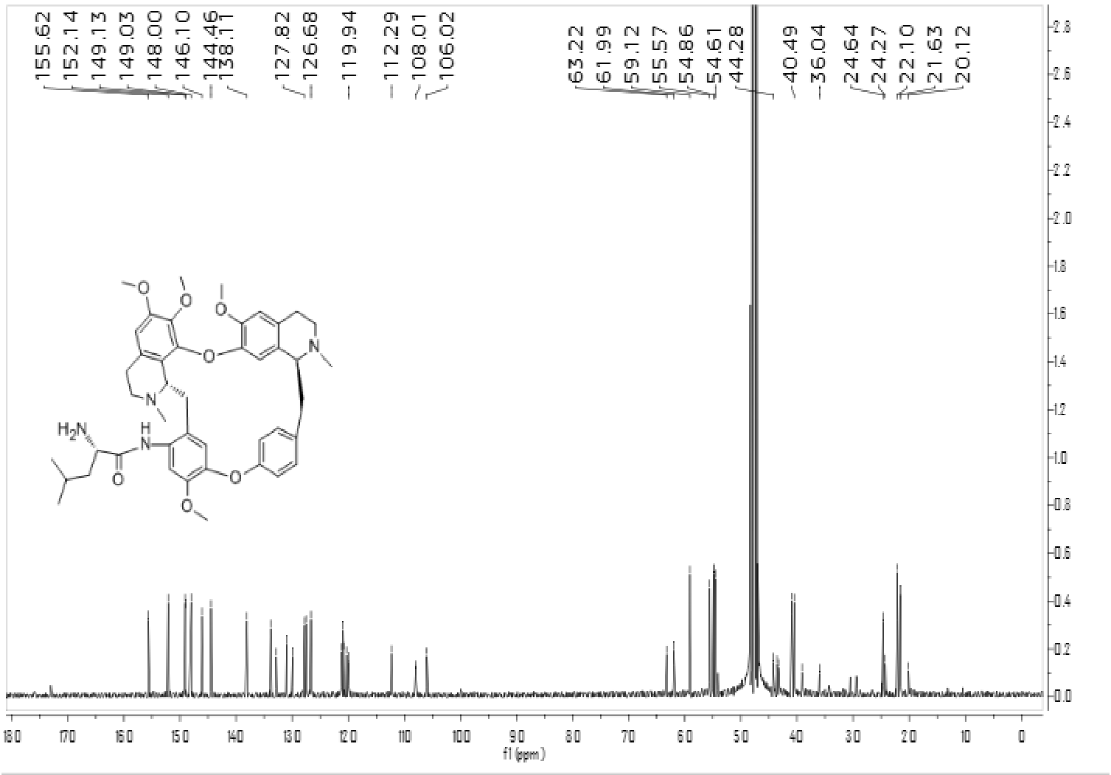

^{13}C NMR spectrum of **15**

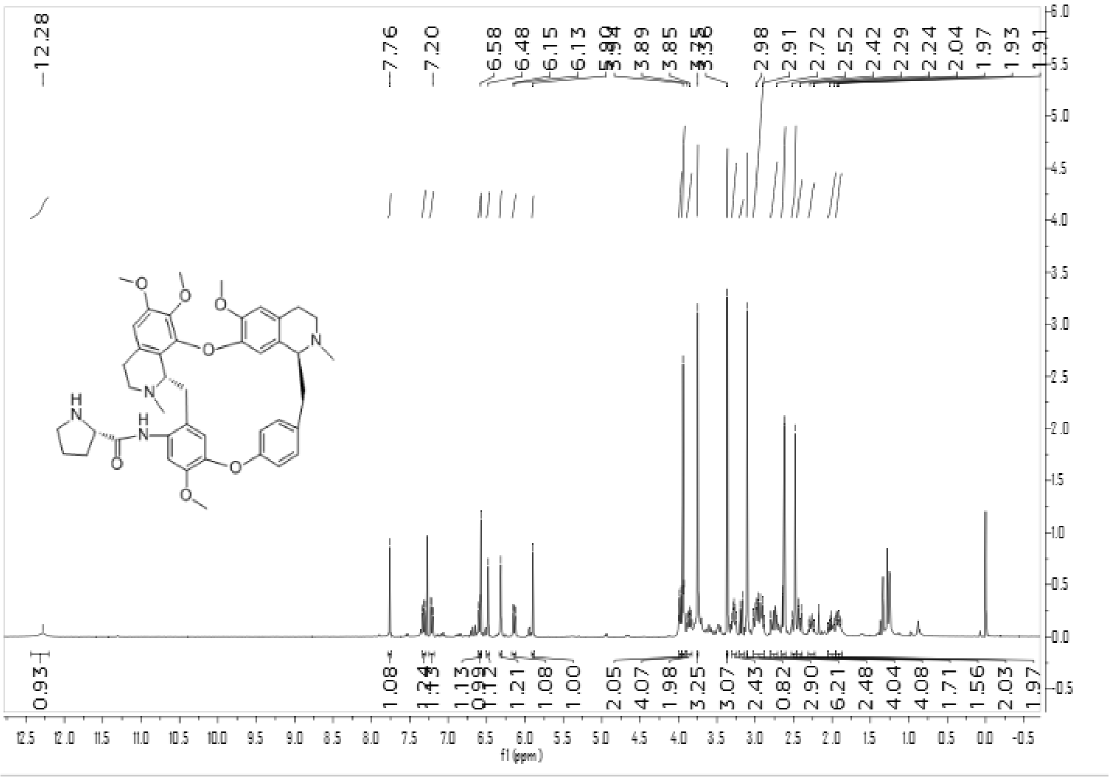

^{1}H NMR spectrum of **16**

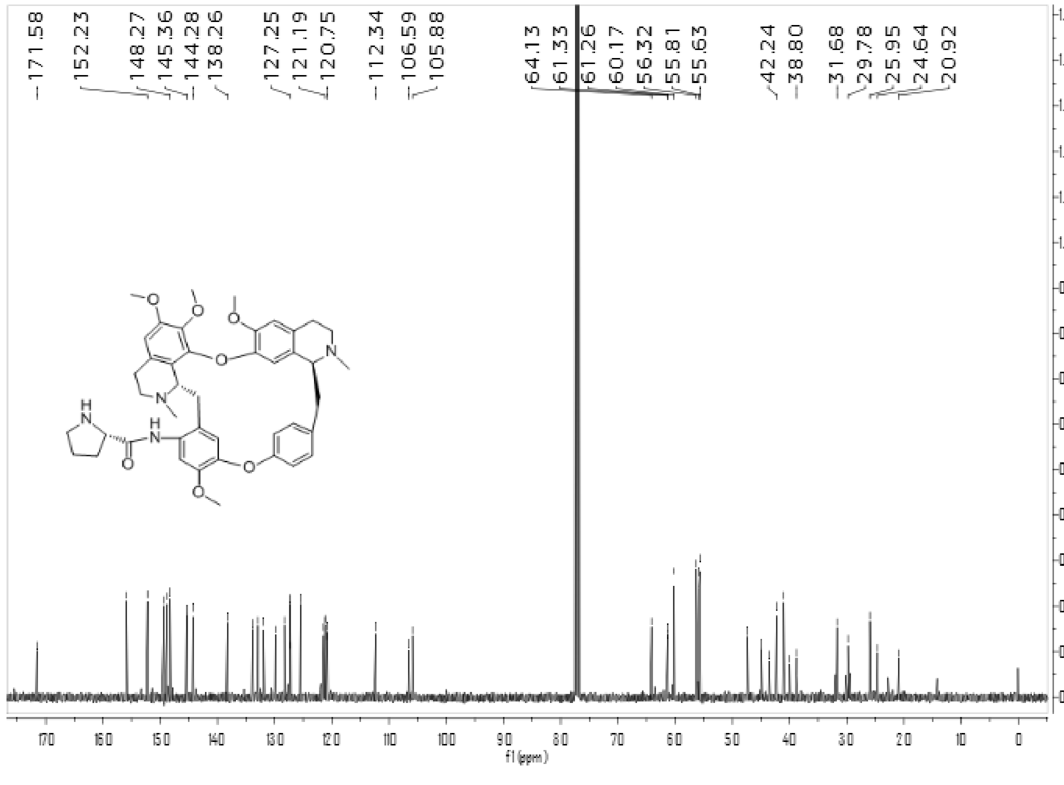

^{13}C NMR spectrum of **16**

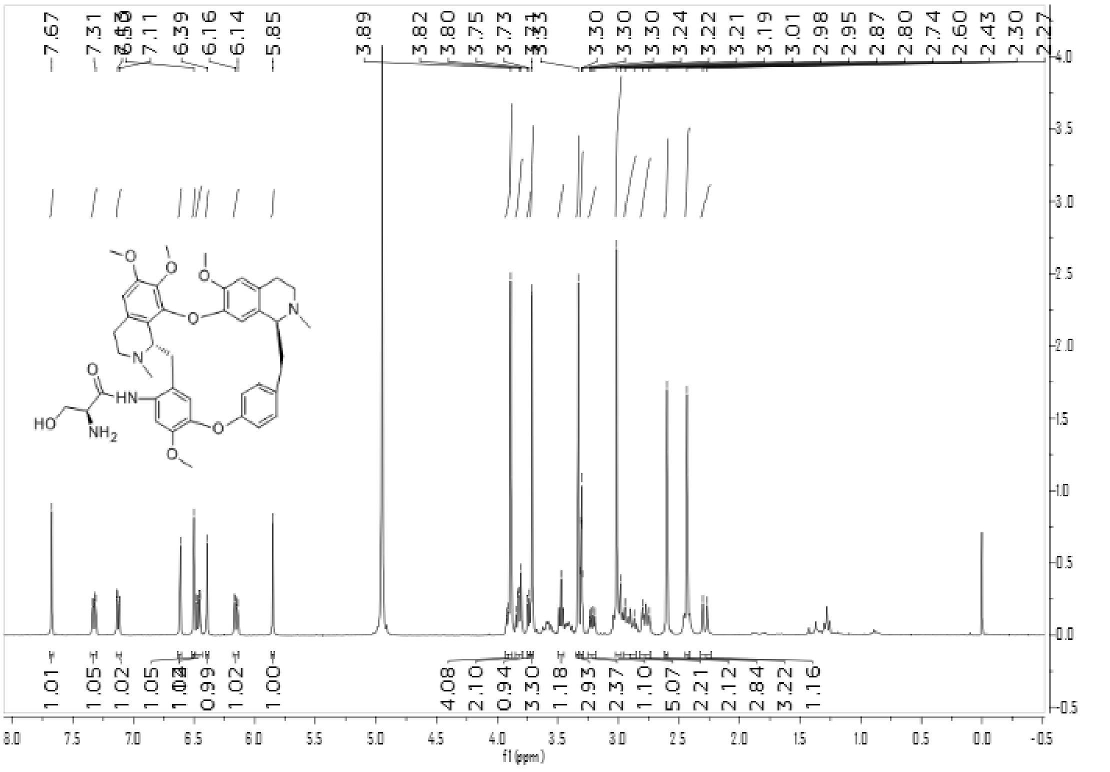

^{1}H NMR spectrum of **17**

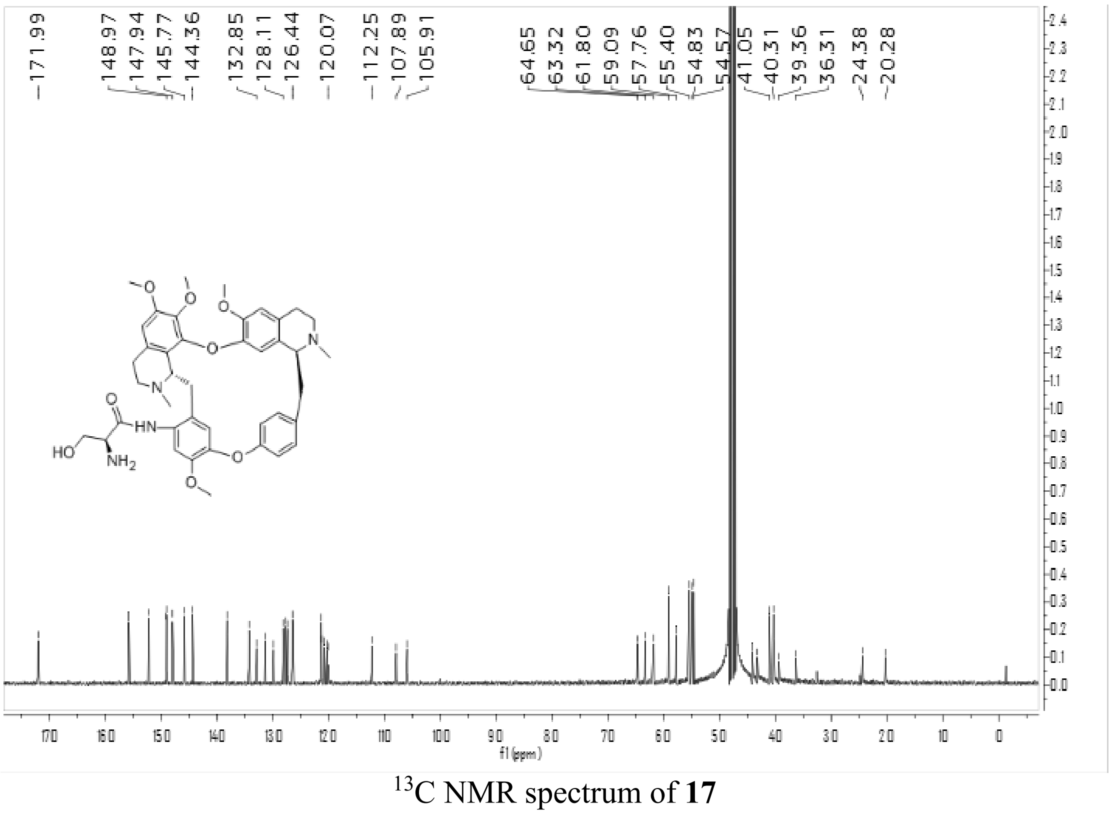

^{13}C NMR spectrum of **17**

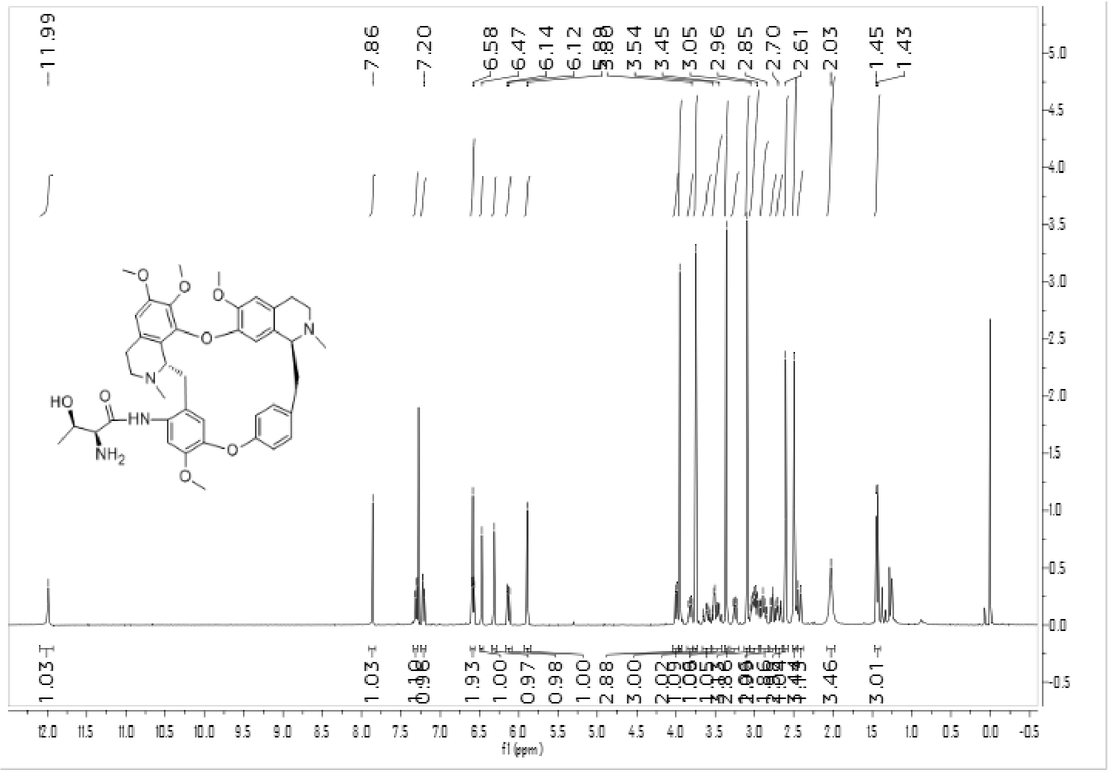

^{1}H NMR spectrum of **18**

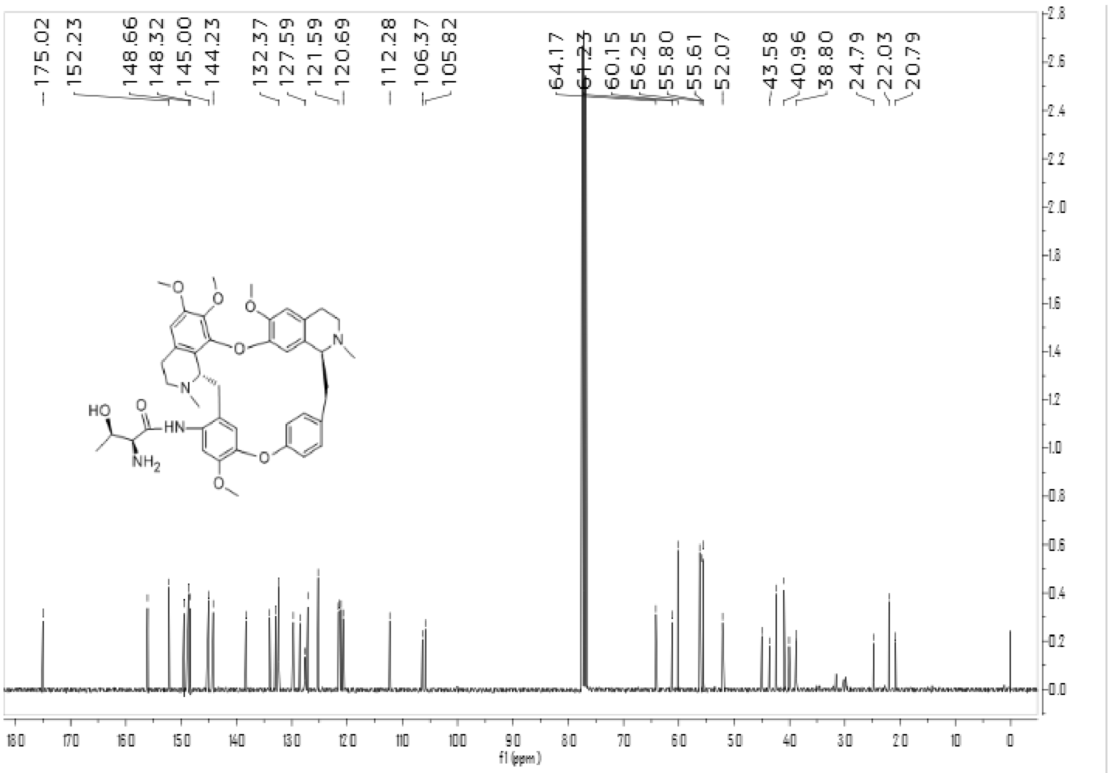

^{13}C NMR spectrum of **18**

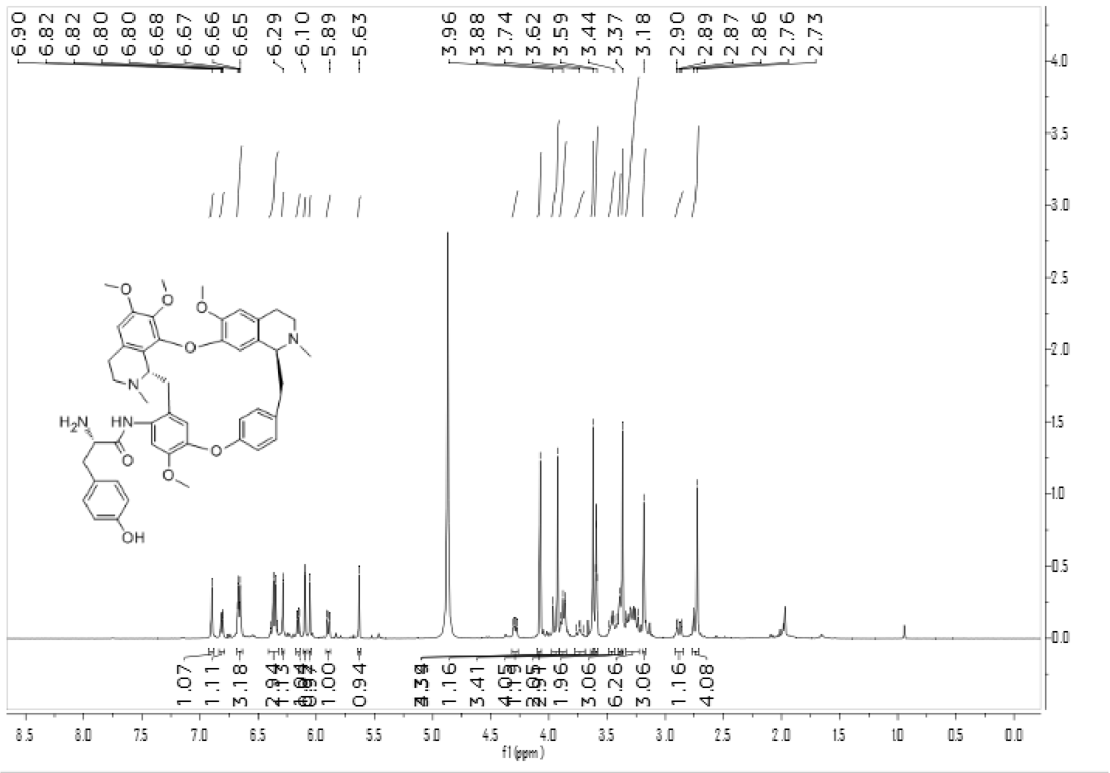

^{1}H NMR spectrum of **20**

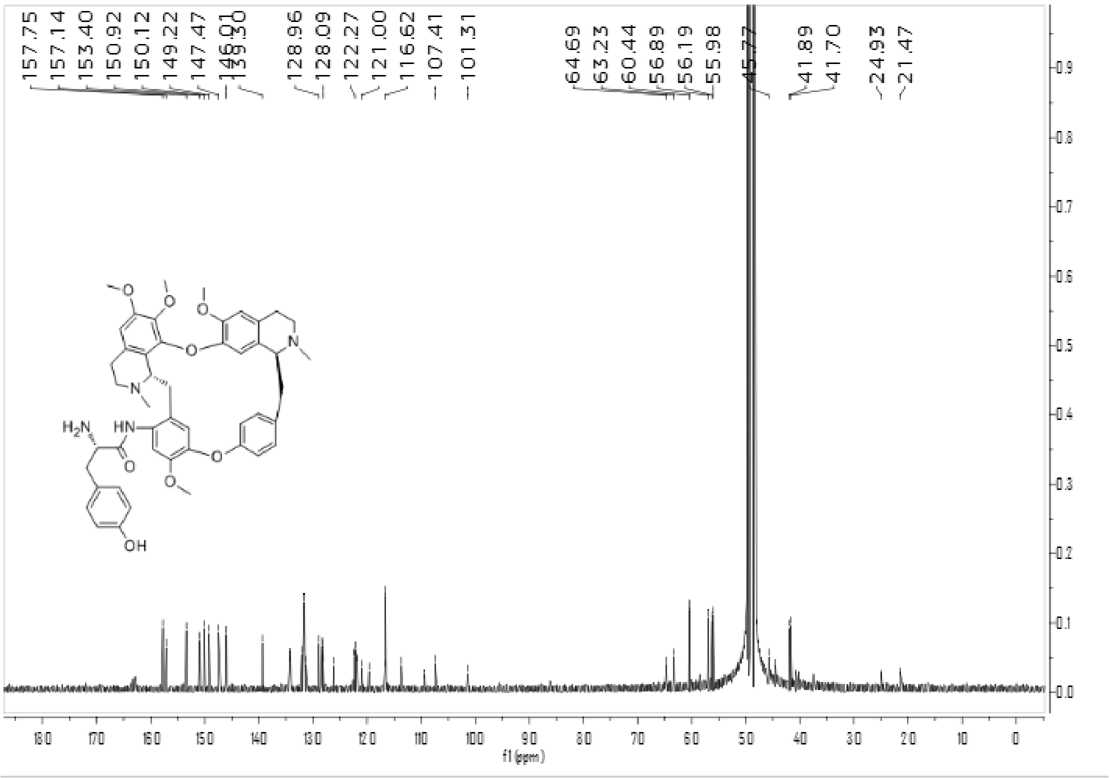

^{13}C NMR spectrum of **20**

IR spectra of compounds

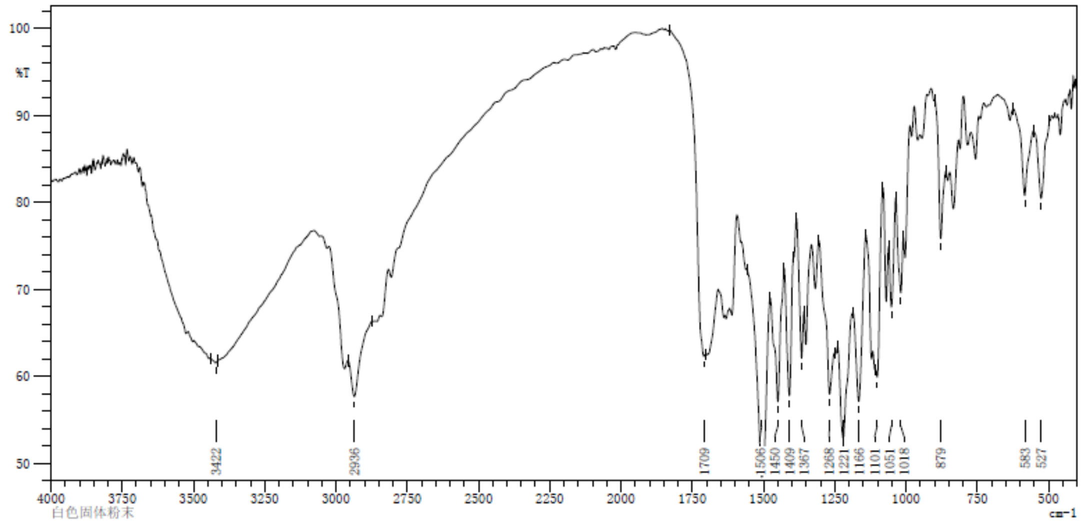

No.	峰	高度	校正强度	基点(高)	基点(低)	面积	校正面积	描述
1	527	80.45	8.31	554	497	832.590	194.919	

2020对外检测（红外） - 1-2658-5 - 3.ispd	3	白色固体粉末	2022/06/09<br>17:43:45

IR spectrum of **3**

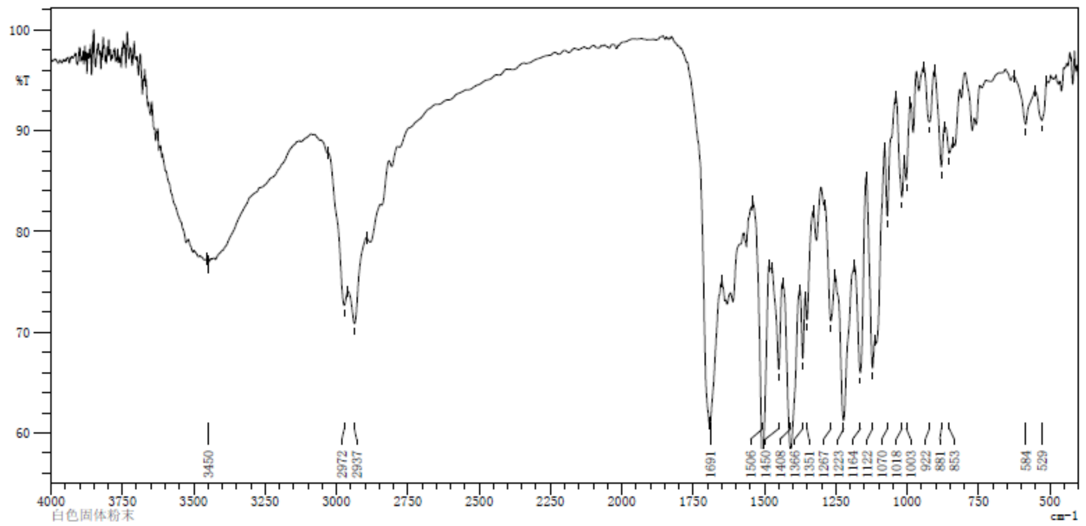

No.	峰	高度	校正强度	基点(高)	基点(低)	面积	校正面积	描述
1	529	91.00	3.51	553	512	304.802	76.780	

2020对外检测（红外） - 1-2654-5 - 7.ispd	7	白色固体粉末	2022/06/09<br>17:11:52

IR spectrum of **7**

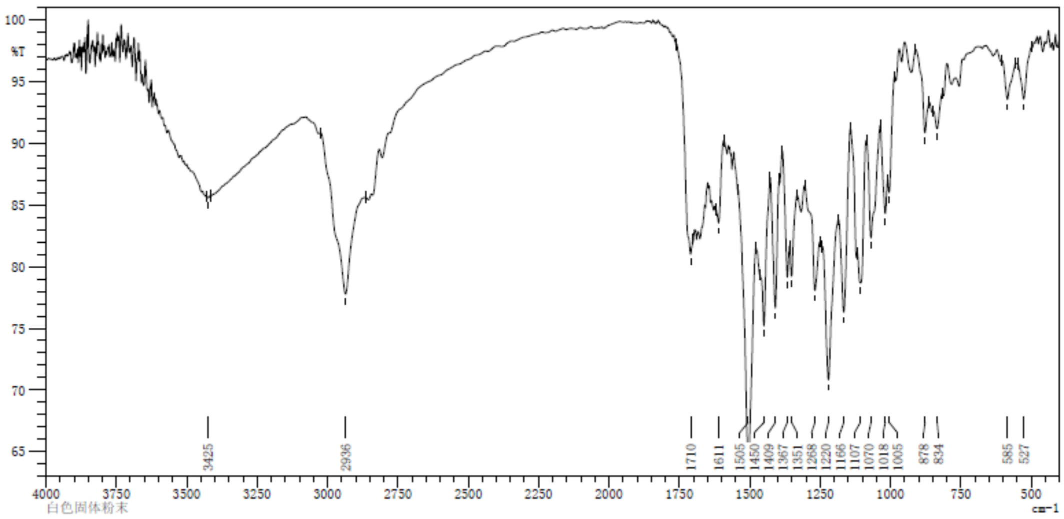

	峰	高度	校正强度	基点(高)	基点(低)	面积	校正面积	描述
1	527	93.57	3.52	550	499	218.484	72.904	

2020对外检测（红外） - 1-2649-5 - 8.ispd	8	白色固体粉末	2022/06/09 16:20:21

IR spectrum of **8**

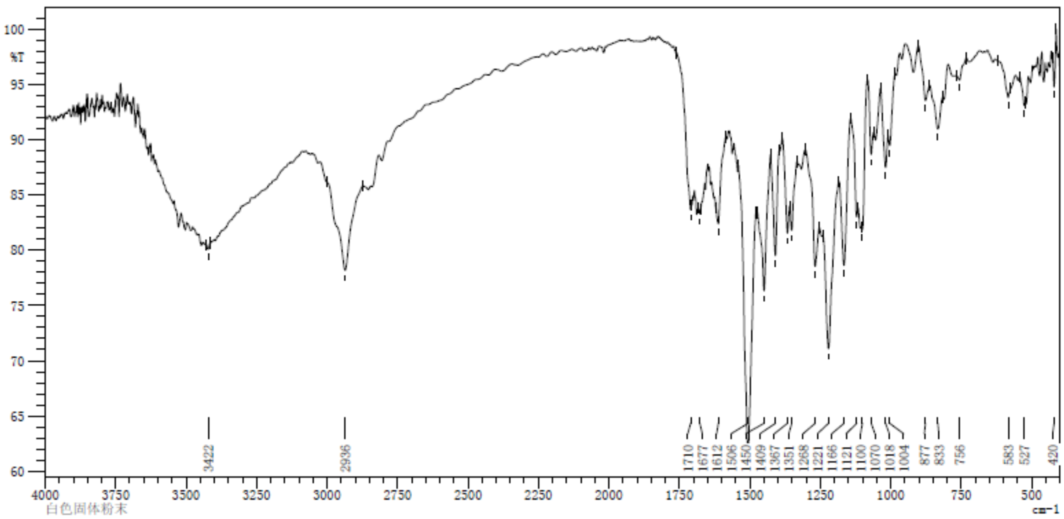

	峰	高度	校正强度	基点(高)	基点(低)	面积	校正面积	描述
1	420	94.76	3.72	425	414	40.579	25.514	

2020对外检测（红外） - 1-2657-5 - 11.ispd	11	白色固体粉末	2022/06/09 17:36:38

IR spectrum of **11**

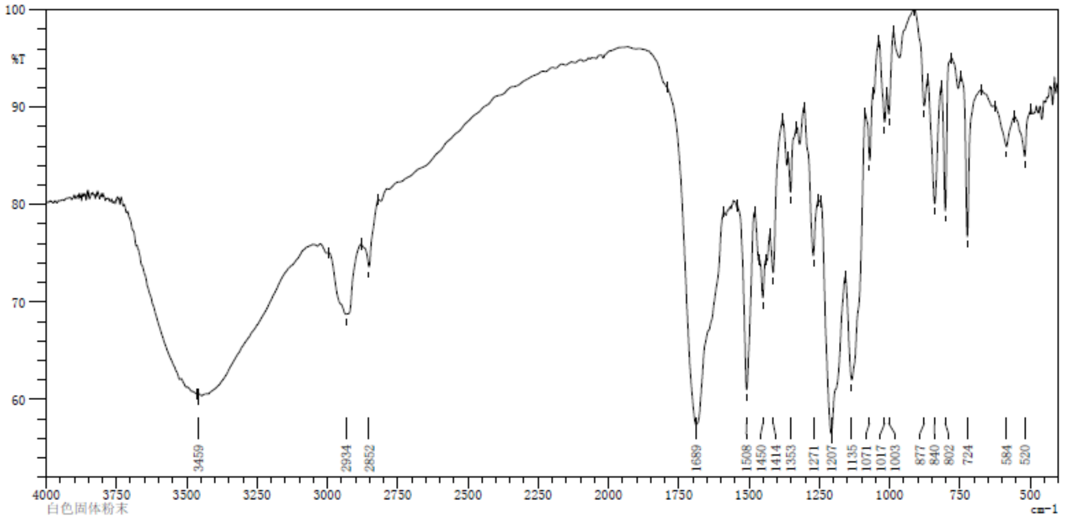

	峰	高度	校正强度	基点(高)	基点(低)	面积	校正面积	描述
1	520	84.95	4.59	556	498	706.718	95.897	

2020对外检测（红外） - 1-2655-5 - 12.ispd	12	白色固体粉末	2022/06/09<br>17:21:43

IR spectrum of **12**

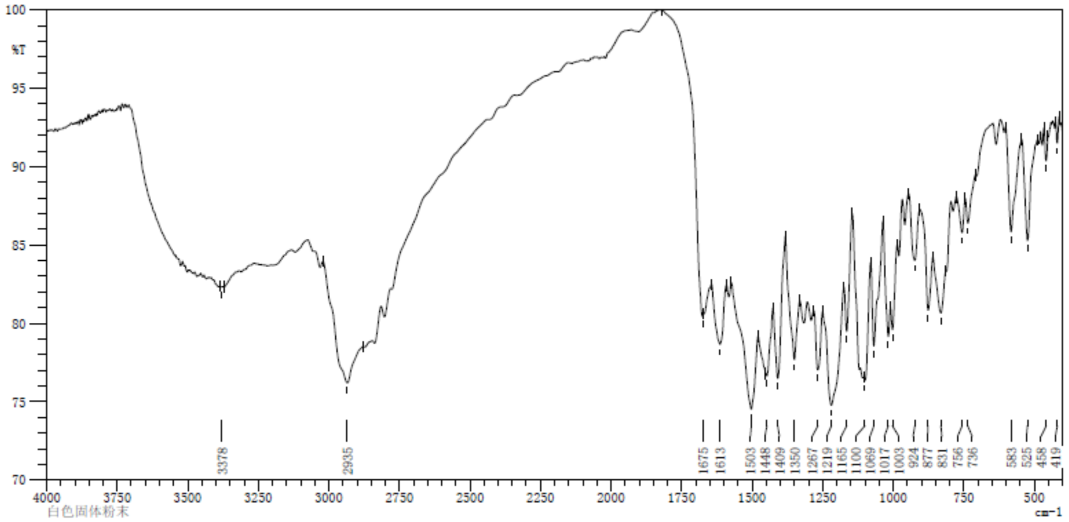

	峰	高度	校正强度	基点(高)	基点(低)	面积	校正面积	描述
1	419	91.50	1.52	424	413	90.731	9.842	

2020对外检测（红外） - 0-31-1 - 14.ispd	14	白色固体粉末	2022/06/09<br>18:01:18

IR spectrum of **14**

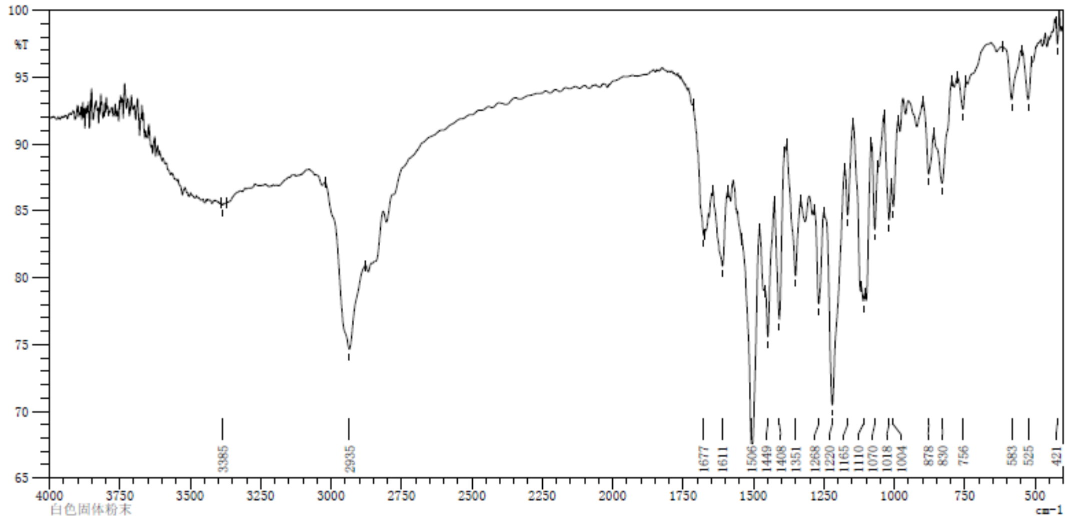

	峰	高度	校正强度	基点(高)	基点(低)	面积	校正面积	描述
1	421	97.48	1.93	425	414	18.636	13.522	

2020对外检测（红外） - 1-2652-5 - 15.ispd	15	白色固体粉末	2022/06/09<br>16:53:12

IR spectrum of **15**

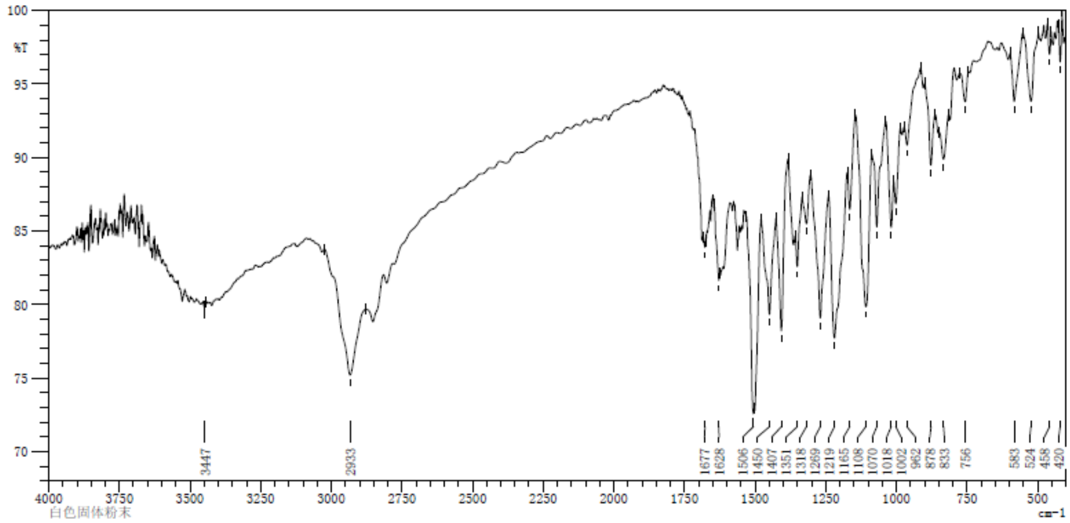

	峰	高度	校正强度	基点(高)	基点(低)	面积	校正面积	描述
1	420	96.50	2.90	425	414	26.016	20.085	

2020对外检测（红外） - 1-2653-5 - 16.ispd	16	白色固体粉末	2022/06/09<br>17:04:01

IR spectrum of **16**

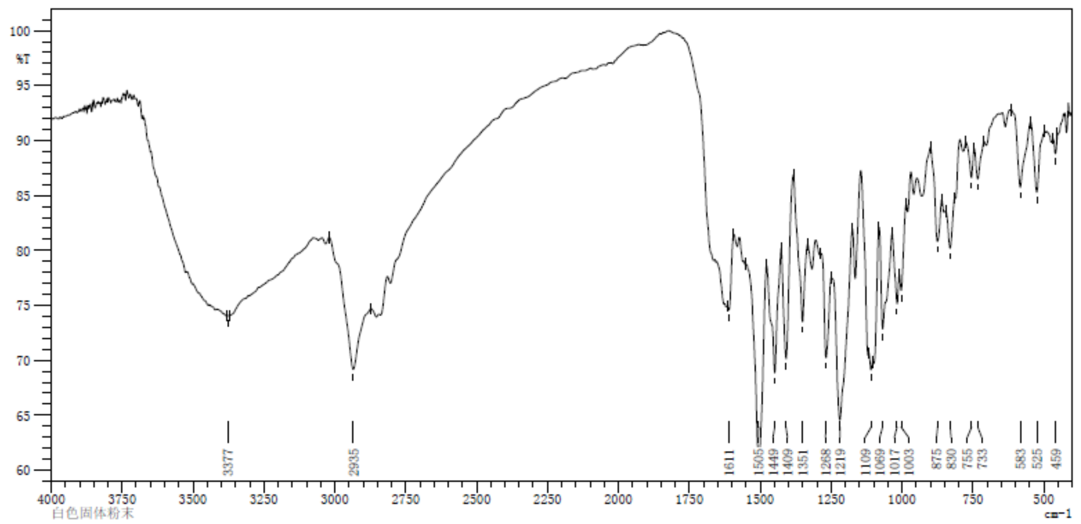

	峰	高度	校正强度	基点(高)	基点(低)	面积	校正面积	描述
1	459	88.80	1.62	468	452	161.187	12.986	

2020对外检测（红外） - 1-2651-5 - 17.ispd	17	白色固体粉末	2022/06/09 16:45:31

IR spectrum of **17**

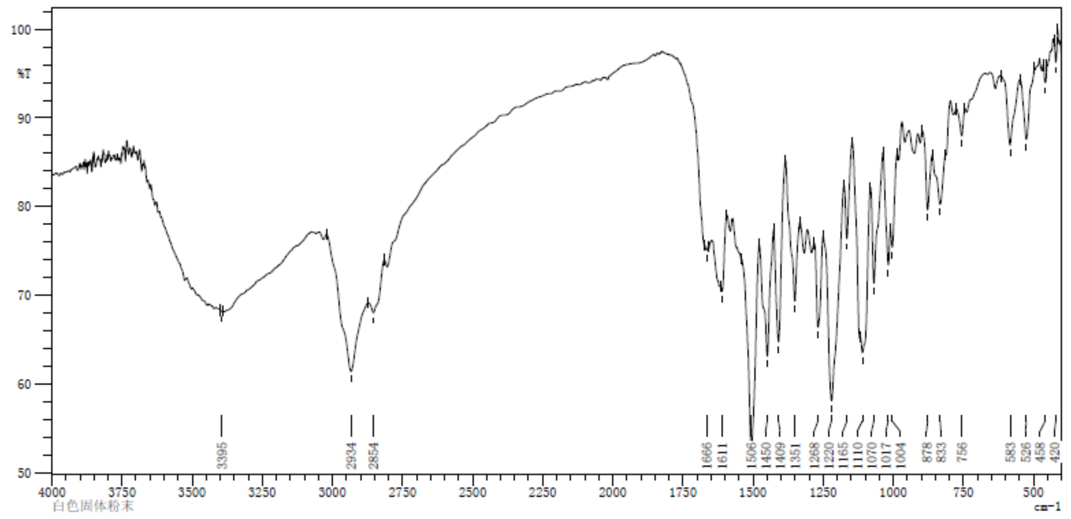

	峰	高度	校正强度	基点(高)	基点(低)	面积	校正面积	描述
1	420	96.27	2.97	425	414	28.195	20.574	

2020对外检测（红外） - 1-2656-5 - 18.ispd	18	白色固体粉末	2022/06/09 17:28:26

IR spectrum of **18**

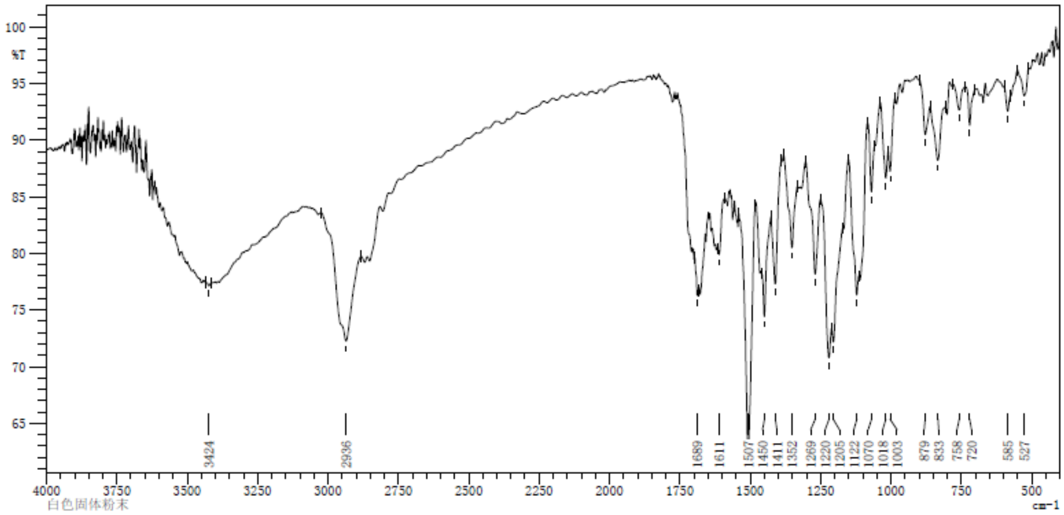

	峰	高度	校正强度	基点(高)	基点(低)	面积	校正面积	描述
1	527	93.91	2.36	551	511	195.001	47.141	

2020对外检测（红外） - 1-2650-5 - 20.ispd	20	白色固体粉末	2022/06/09<br>16:31:35

IR spectrum of **20**
